# IgG1 memory B cells keep the memory of IgE responses

**DOI:** 10.1038/s41467-017-00723-0

**Published:** 2017-09-21

**Authors:** Jin-Shu He, Sharrada Subramaniam, Vipin Narang, Kandhadayar Srinivasan, Sean P. Saunders, Daniel Carbajo, Tsao Wen-Shan, Nur Hidayah Hamadee, Josephine Lum, Andrea Lee, Jinmiao Chen, Michael Poidinger, Francesca Zolezzi, Juan J. Lafaille, Maria A. Curotto de Lafaille

**Affiliations:** 1 Singapore Immunology Network (SIgN), 8A Biomedical Grove, Singapore, 138648 Singapore; 2 School of Biological Sciences, Nanyang Technological University, 60 Nanyang Drive, Singapore, 637551 Singapore; 3 Division of Pulmonary, Critical Care and Sleep Medicine, Departments of Medicine and Cell Biology, New York University School of Medicine, 550 First Ave, New York, 10016 USA; 4 Galderma R&D, Les Templiers, 2400 route des Colles, Sophia Antipolis, 06410 Biot France; 5 Skirball Institute and Department of Pathology, New York University School of Medicine, 540 First Ave, New York, 10016 USA

## Abstract

The unique differentiation of IgE cells suggests unconventional mechanisms of IgE memory. IgE germinal centre cells are transient, most IgE cells are plasma cells, and high affinity IgE is produced by the switching of IgG1 cells to IgE. Here we investigate the function of subsets of IgG1 memory B cells in IgE production and find that two subsets of IgG1 memory B cells, CD80^+^CD73^+^ and CD80^−^CD73^−^, contribute distinctively to the repertoires of high affinity pathogenic IgE and low affinity non-pathogenic IgE. Furthermore, repertoire analysis indicates that high affinity IgE and IgG1 plasma cells differentiate from rare CD80^+^CD73^+^ high affinity memory clones without undergoing further mutagenesis. By identifying the cellular origin of high affinity IgE and the clonal selection of high affinity memory B cells into the plasma cell fate, our findings provide fundamental insights into the pathogenesis of allergies, and on the mechanisms of antibody production in memory B cell responses.

## Abstract (web summary)

IgE is an important mediator of protective immunity as well as allergic reaction, but how high affinity IgE antibodies are produced in memory responses is not clear. Here the authors show that IgE can be generated via class-switch recombination in IgG1 memory B cells without additional somatic hypermutation.

## Introduction

IgE antibodies that bind allergens with high affinity are capable of mediating life-threatening anaphylaxis. IgE antibodies are the least abundant in serum, and display the lowest serum half-life of all immunoglobulins^1^. Distinctive features of the differentiation of IgE^−^-producing cells limit the production of high affinity IgE. IgE germinal centre (GC) cells are transient and highly apoptotic, and do not give rise to functional IgE memory B cells (MBC) or high affinity IgE plasma cells (PC)^2–4^. Class switching of antigen-specific IgG1 cells to become IgE cells, known as sequential switching, has been proposed as the mechanism involved in the production of affinity-matured IgE antibodies in memory responses^5–7^, but the extent by which sequential switching compensates for the lack of functional IgE MBC is not known. Mice deficient in class-switch recombination to IgG1 display profoundly impaired affinity maturation of IgE antibodies^5^. IgE is still produced via direct class switching of IgM to IgE but other IgGs or IgA do not contribute to the IgE response.

Previous studies showed that IgE production in secondary responses required CD4 T cell help and the cytokine Interleukin (IL)-4, while production of IgG1 was independent of IL-4^8, 9^. These findings suggested a need for de novo class-switch recombination to IgE in memory responses and implicated a lack of memory IgE cells. By contrast the IL-4^−^ independence of IgG1 production in memory responses indicated that there were memory IgG1 cells that differentiated into IgG1 plasma cells, a process that is independent of IL-4^4, 10^. It was later shown that IgE^–^B220^+^ B cells, but not IgE^+^B220^+^ B cells, mediate IgE production in secondary responses^3^, consistent with the observed lack of functional IgE memory cells^2–4, 11^.

Published work supports a function for human IgG cells in generating IgE-producing cells. First, studies of allergen reactivity demonstrated production of allergen-specific IgG1, IgG4 and IgE in allergic individuals, indicative of a co-ordinated regulation of these immunoglobulin isotypes^12^. Second, sequencing of the Sμ-Sε switch regions of human IgE genes found Sγ switch region repeat remnants indicative of sequential switching^13–16^. Third, repertoire analysis of the rearranged immunoglobulin genes in human peripheral blood B cells identified common lineages between IgG1 and IgE^17^, suggesting a parental-progeny relationship.

Memory antibody responses typically produce a fast increase in the levels and affinity of antigen-specific antibodies^18–20^. It is not known if this process involves new somatic mutation, or if it results from the selective expansion and differentiation of pre-existing memory clones. Mouse MBC are heterogeneous in their origins, phenotypes and functions^21^. MBC may express IgM, IgG or IgA, and be of GC or extra-GC origin^20, 22, 23^. It was reported that IgG1 MBC preferentially differentiate into PC upon activation, while IgM MBC give rise to secondary GC^18, 24^. Studies also identified a subpopulation of IgG1 MBC that preferentially forms PC upon activation, and another that forms GC cells^25^, but their function in IgE responses remains unknown.

Here, we demonstrate the ability of IgG1 MBC to generate IgE-producing cells in secondary responses. We found that the IgG1 MBC subset with a phenotype that is ‘pro-PC’ gives rise to an early burst of high affinity IgE that mediates anphylaxis, while another ‘pro-GC’ subset generates a late, low affinity and non-pathogenic IgE response. Thus, the contribution of all subsets of IgG1 MBC to IgE potentially modulates the overall IgE pathogenicity. Repertoire analyses of parental MBC and their progenies using high throughput RNA sequencing demonstrated that high affinity clones present at low frequency in the pro-PC MBC subset form the expanded repertoire of high affinity clones in their progeny. These results support a model in which high affinity PC clones are derived from the selection and expansion of rare high affinity IgG1 MBC clones without further mutagenesis. Our findings thus help enhance the understanding of general mechanisms of production of antigen-specific antibodies, as well as the generation and pathogenicity of IgE antibodies.

## Results

### CD80^+^CD73^+^ IgG1 MBC generate high affinity IgE PC

The ability of subsets of mouse IgG1 MBC to give rise to IgE-secreting cells was analysed using well characterised models of type 2 responses: infection of BALB/c mice with the helminth parasite *Nippostrongylus brasiliensis* (*N.b*.), and immunisation of TB monoclonal (TBmc) mice^6, 26^. TBmc mice carry monospecific populations of CD4 T cells specific for chicken ovalbumin (OVA) and of B lymphocytes specific for an influenza virus hemagglutinin peptide (HA). PEP1 is a mutant variant of HA that displays greatly decreased binding to naive B cells from TBmc mice and is used to trace antibody affinity maturation^6^.

**Fig. 1:**
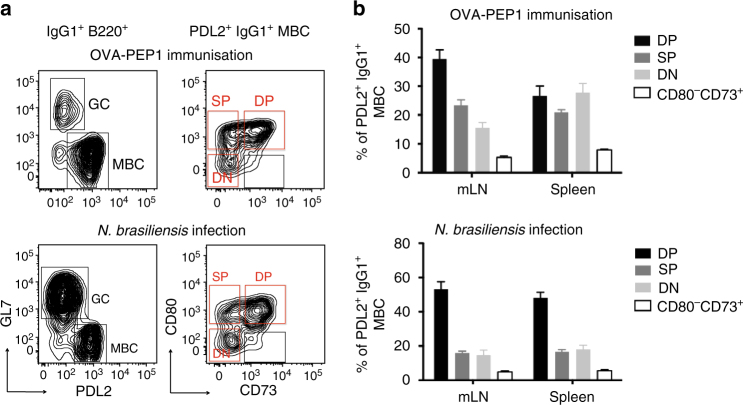
Diversity of IgG1 memory B cells (MBC) in type 2 responses. **a**, **b** IgG1^+^ MBC subsets in spleen and mesenteric LN (mLN) of TBmc mice immunised with OVA-PEP1 (OVA-PEP1 immunisation), and of BALB/c mice infected with *Nippostrongylus brasiliensis* (*N.brasiliensis* infection) at 10 weeks after treatment. The samples in each group were pooled, pre-enriched by depletion of IgD^+^, CD138^+^ and CD3^+^ cells. **a** Frequency of GL7^+^ germinal centre (GC) cells and PDL2^+^ MBC among gated IgG1^+^ B220^+^ cells (*left*), and identification of CD73^+^CD80^+^ (DP), CD73^–^CD80^+^ (SP) and CD73^−^CD80^−^ (DN) IgG1 MBC subsets (*right*). **b** Frequency of CD73^+^CD80^+^ (DP), CD73^–^CD80^+^ (SP), CD73^−^CD80^–^ (DN) and CD73^+^CD80^–^ cells among gated PDL2^+^IgG1^+^ MBC from OVA-PEP1 immunised TBmc mice (*top*) or *N.brasiliensis* infected BALB/c mice (*bottom*). Data are mean ± SEM of 5 OVA-PEP1 immunisation experiments and six *N.brasiliensis* infection experiments

We first characterised the different types of IgG1 cells in lymphoid organs of immunised or infected mice. To identify IgG1 GC cells and IgG1 MBC subsets, B cells were first pre-enriched by depletion of CD3e^+^, IgD^+^, CD138^+^ and TER-119^+^ cells, and were then stained with antibodies to IgG1, B220, PDL2, GL7, CD138, CD73 and CD80^25, 27^. B cells were identified by B220 staining in a CD4^−^ single cell/lymphocyte gate (Supplementary Fig. 1a for gating strategy). Within the B220^+^ population, MBC were identified as PDL2^+^GL7^−^, distinguishing them from late GC cells (PDL2^−^GL7^+^) (Fig. 1a). Expression of CD73 and CD80 revealed three main PDL2^+^IgG1^+^ MBC populations: CD73^+^CD80^+^ double positive (DP), CD73^−^CD80^+^ single positive (SP), and CD73^−^CD80^−^ double negative (DN) cells (Fig. 1a). Notably, there were more GC cells in *N.b*.-infected BALB/c mice than in immunised TBmc mice at week 10 (Fig. 1a), and DP IgG1 MBC were found at a higher frequency among MBC in infected than immunised mice (Fig. 1b). As expected from previous studies, very few IgE GC cells remained by this time, B220^+^-IgE^+^ memory B cells were practically undetectable, while there were still CD138^+^IgE^+^ PC and CD138^+^IgG1^+^ PC in lymphoid organs (Supplementary Fig. 1b).

**Fig. 2:**
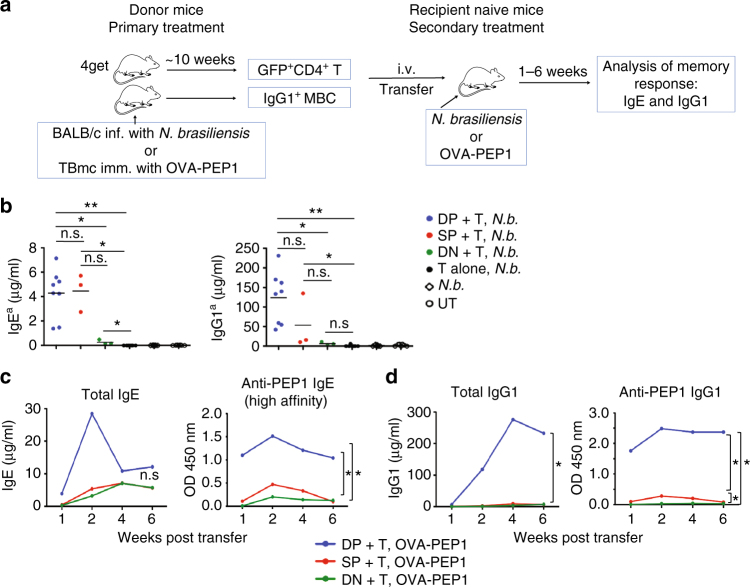
CD73^+^CD80^+^ IgG1 MBC give rise to high-affinity IgE. **a** Diagram of adoptive transfer experiments to evaluate IgE production from IgG1 MBC subsets (i.v.: intravenously). **b** Donor-derived IgE^a^ and IgG1^a^ production in CB17 *IgH*^*b*^ recipient mice transferred with IgG1^+^ MBC (DP, SP and DN) and CD4 memory T cells (T) from *N.brasiliensis (N.b.)* infected mice, and subsequently infected with *N.b*. IgE^a^ and IgG1^a^ serum levels were determined at 2 weeks after transfer and infection. The data shows average of *n* = 8 for DP + T, *n* = 3 for SP + T, *n* = 3 for DN + T, or *n* = 7 for T alone, *n* = 7 for *N.b*. and *n* = 7 for untreated (UT) groups. The samples were pooled from three independent experiments. Statistical analysis was performed using Mann–Whitney–Wilcoxon *U*-test. *P < 0.05, **P < 0.01; n.s., not significant (*P* > 0.05). **c**, **d** Kinetics of the production of total and PEP1-specific IgE **c** and IgG1 **d** in serum of *Rag1* KO mice that were transferred with IgG1 MBC (DP, SP and DN) and CD4 memory T cells (T) from OVA-PEP1 immunised TBmc mice, and were subsequently immunised with OVA-PEP1. The data for each time point was obtained with pooled serum from 4 (DP and SP) and 3 (DN) recipient mice per group. The serum was diluted 500-fold to measure PEP1-specific IgG1, and 100-fold to measure PEP1-specific IgE. Non-parametric Kruskal–Wallis rank-sum test was used to calculate *P* value. *P < 0.05; n.s., not significant (*P* > 0.05). The data are representative of three independent experiments

To better understand the genesis of memory IgE responses, we tested the ability of each subset of IgG1 MBC to give rise to IgE-producing cells. PDL2^+^ IgG1 MBC subsets were sorted into DP, SP and DN subsets based on expression of CD73 and CD80. Subset identity, as well as lack of contaminating IgE cells, were confirmed by flow cytometry and RNA/cDNA expression analysis (Supplementary Fig. 2a–c). To ensure appropriate T cell help for class-switch recombination to IgE, IL-4-competent CD4 memory T cells were isolated as CD4^+^GFP^+^ cells from 4get mice^28^ (Supplementary Fig. 2d). 4get mice carry an internal ribosomal entry site and the green fluorescent protein (*IRES-GFP)* gene in the 3′ untranslated region of the *Il4* gene, thus reporting *Il4* transcription through GFP expression. Purified IgG1 MBC and CD4 memory T cells were used in adoptive transfer experiments (Fig. 2a).

DP, SP and DN IgG1 MBC and CD4 memory T cells were sorted from *N.b.-*infected BALB/c mice and transferred into CB17 mice (*IgH*^*b*^ haplotype on BALB/c background) that were then infected with *N.b*. Two weeks later, donor-derived IgE^a^ and IgG1^a^ were measured in the recipient mice (Fig. 2b). IgE^a^ and IgG1^a^ antibodies were detected in mice transferred with DP IgG1 MBC at levels significantly higher than in mice transferred with DN IgG1 MBC, consistent with the described ability of CD73^+^PDL2^+^IgG1 MBC to differentiate into plasma cells^25^. No statistical significant differences in serum IgE^a^ or in serum IgG1^a^ were observed between mice transferred with DP IgG1 MBC or SP IgG1 MBC. Serum IgE^a^ levels were detected above background (T alone, *N.b*.) in all groups transferred with IgG1 MBC (DP, SP and DN) indicating that all IgG1 MBC have the potential to contribute to serum IgE.

To determine the affinity of IgE derived from the different IgG1 MBC populations, we used the TBmc model of immunisation with OVA-PEP1^6^. Ten weeks after primary immunisation, IgG1 MBC subsets and CD4 memory T cells were isolated and transferred to *Rag1* knockout (KO) mice, and the recipient mice were immunised with OVA-PEP1. Serum antibodies were analysed at weeks 1, 2, 4 and 6.

IgE appeared and peaked earlier in mice transferred with DP IgG1 MBC, and only the DP subset gave rise to PEP1-specific high affinity IgE (Fig. 2c). IgE was produced in mice transferred with SP or DN IgG1 MBC with slow kinetics. The SP- and DN-derived IgE was of low affinity, reaching about half the level found in DP-derived IgE (Fig. 2c). Strikingly, IgG1 antibody levels were much higher in mice transferred with DP IgG1^+^ MBC than in the other two groups (Fig. 2d).

PEP1-specific antibodies in OVA-PEP1 immunised TBmc mice indicate affinity maturation of the antibody response^6^. In contrast, HA-binding antibodies in these mice are non-affinity matured, since the critical CDR3 residues for HA-binding^29, 30^ are the ones mutated in PEP1-binding antibodies. HA-specific IgE levels in mice transferred with DP, SP or DN IgG1 MBC were comparable among groups at 6 weeks (Supplementary Fig. 3a, left). In contrast, the HA-specific IgG1 level was much higher in mice transferred with DP IgG1 MBC, (Supplementary Fig. 3a, right) as shown by total IgG1 measurement (Fig. 2d). Similar results were obtained when CD73^+^ IgG1 MBC (corresponding to DP IgG1 MBC) and CD73^−^ IgG1 MBC (corresponding to SP plus DN IgG1 MBC) from OVA-PEP1 immunised TBmc mice were adoptively transferred into wild-type (WT) BALB/c mice (Supplementary Fig. 3b).

Collectively, these results demonstrate that CD73^+^ DP IgG1 MBC are the precursors of IgE cells producing high affinity IgE in memory responses, while SP and DN IgG1 MBC give rise to a late, low affinity IgE response.

### Hierarchical affinity of the IgG1 and IgE from DP IgG1 MBC

To correlate the observed differences in IgE affinity with the presence of specific B cell receptor (BCR) mutations, we determined the repertoire of VDJ heavy chain sequences of parental donor IgG1 MBC and their IgE and IgG1 derived cells (referred as IgE and IgG1 progenies of IgG1 MBC) using next generation sequencing. Samples utilised for sequencing contained roughly equivalent number of cells (between 1.3 × 10^5^ and 1.5 × 10^5^). Read quality values indicated high quality sequencing, and rarefaction curves demonstrated sufficient sequencing depth (Supplementary Fig. 4a–d). Libraries of nucleotides and amino acid sequences were derived for each sample to determine the frequency and type of somatic mutations.

Three CDR3 amino acids mutations (R97T, N100aS/T and A101T) contribute to high affinity binding to PEP1 in TBmc mice^6^. Repertoire analysis showed that all three donor IgG1 MBC subsets had less mutated VDJ genes than IgG1 GC cells (Supplementary Fig. 5a–c), as described^18–20^. Among MBC, DP IgG1 were slightly more mutated than SP or DN IgG1 MBC, but high affinity mutations were found in all subsets.

**Fig. 3:**
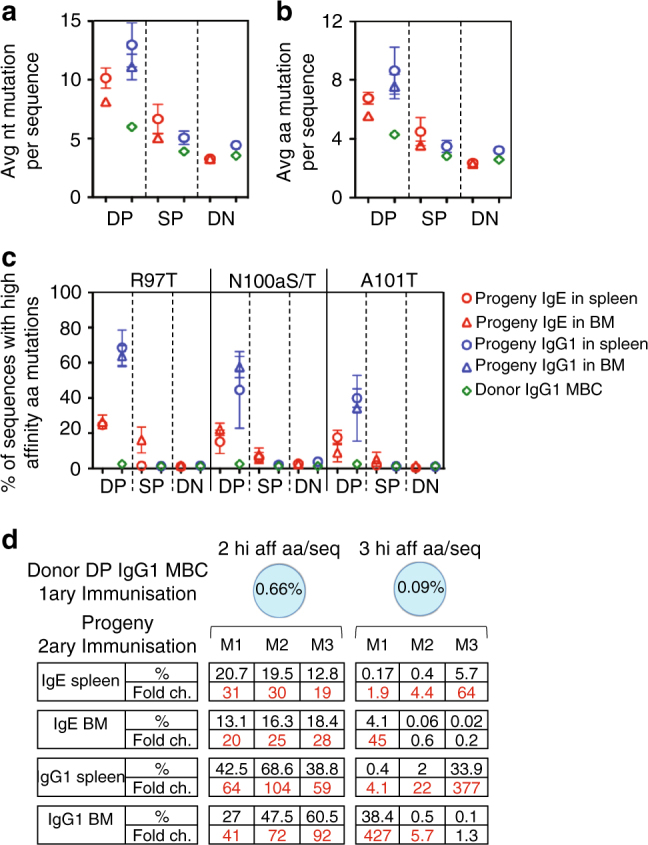
PC derived from DP IgG1 MBC are enriched in high affinity mutations. VDJ nucleotide (nt) and amino acid (aa) sequences were compared between IgG1 MBC subsets and their IgE and IgG1 progeny cells. IgG1 MBC subsets were purified from 10 weeks OVA-PEP1 immunised TBmc mice, and transferred together with CD4 memory T cells into *Rag1* KO recipient mice. The recipient mice were immunised with OVA-PEP1. Spleen and bone marrow (BM) of the recipient mice were collected 2 weeks later. The VDJ repertoires of parental IgG1 MBC subsets and their IgG1 and IgE progeny were analysed using next generation sequencing. **a**, **b** Average number of nt **a** and aa **b** mutations per sequence. **c** Percentage of sequences containing R97T, N100aS/T or A101T high affinity CDR3 mutations. **a**–**c** Data are average ± SEM of three mice per group. **d** Enrichment in the percentage of CDR3 sequences containing 2 or 3 high affinity aa per sequence (hi aff aa/seq) in the IgE and IgG1 progeny of DP IgG1 MBC (M1, M2 and M3 indicate three recipient mice). Data are representative of two independent experiments

Comparative analysis of the VDJ sequences of each donor IgG1 MBC subpopulation with their IgE and IgG1 progenies, resulted in several informative findings. First, VDJ genes in the IgE and IgG1 progenies of DP IgG1 MBC had more nucleotide and amino acid mutations, including more high affinity mutations, than the donor DP population. Second, IgE and IgG1 derived from DP IgG1 MBC had more mutations than the progenies of the SP or DN IgG1 MBC. Third, among the progeny of DP IgG1 MBC, IgG1 cells consistently carried more mutations than IgE cells (Fig. 3a–c and Supplementary Figs 6, 7).

The enrichment in high affinity mutations from donor DP IgG1 MBC to progeny cells is exemplified in Fig. 3d. CDR3 sequences carrying 2 high affinity mutations comprised 0.66% of CDR3 sequences in the donor DP IgG1 MBC, but increased by 19–31-fold in the IgE progeny, and by 20-fold to 104-fold in the IgG1 progeny (Fig. 3d).

In summary, there was a marked increase in affinity during the differentiation of DP IgG1 MBC cells into PC, and the establishment of a remarkable hierarchy of affinity of IgG1 antibodies over IgE antibodies.

### Clonal selection of rare high affinity DP IgG1 MBC

**Fig. 4:**
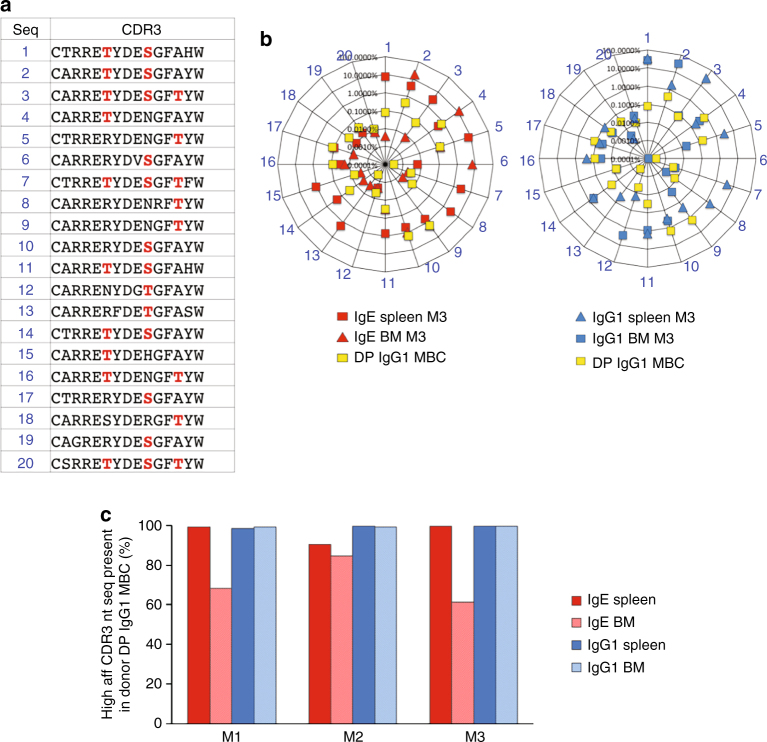
High affinity PC are selected from pre-existent memory clones. The repertoires of VDJ sequences from donor IgG1 MBC subsets (from 10 weeks OVA-PEP1 immunised TBmc mice) and their IgG1 and IgE progenies in spleen and BM of recipient mice (2 weeks after transfer/immunisation), were used to investigate the precursor-progeny relationship. **a** In silico derived sublibrary of the top 20 (frequency descending) VDJ CDR3 aa sequences containing high affinity mutations (in *red*). **b** Radar charts show the presence and frequency of each of the top 20 high affinity CDR3 sequences in donor DP IgG1 MBC and in the progeny IgE (*left*) and IgG1 (*right*) libraries. **a**, **b** The data are from one representative mouse of three recipient mice. **c** Percentage of the progeny IgE and IgG1 nucleotide sequences (nt seq) encoding high affinity CDR3 that were found in the donor DP IgG1 MBC. M1– M3 indicate individual recipient mice

The rapid appearance of high affinity PC could be explained by selective clonal expansion of DP memory precursors, and/or by additional somatic mutation and selection of memory clones. To address this, we established clonal relationships using the VDJ repertoire data. From a library of CDR3 amino acid sequences identified in DP donor IgG1 MBC and progenies containing sequences with 0.01% or higher frequency in at least one sample, we identified 35 CDR3 sequences that contain high affinity mutations (R97T, N100aS/T and A101T^6^; Supplementary Table 1). Out of the 35 sequences in the recipient mice, all were found in the progeny IgE data sets, 32 were found in the progeny IgG1 dataset, and 33 in the precursor DP IgG1 MBC. To better illustrate these findings, the presence and frequency of the 20 more frequent high affinity CDR3 clones in DP donor cells and their progeny in one representative mouse is shown (Fig. 4a, b). All 20 high affinity CDR3 clones (Fig. 4a) were present in the donor DP IgG1 MBC and in their IgE and IgG1 progenies (Fig. 4b). Furthermore, donor and progeny VDJ sequences sharing the same CDR3 amino acid sequence tended to share the same CDR2 and CDR1 sequences (Supplementary Fig. 8).

Clonal relationships were analysed at the nucleotide level. All nucleotide-encoding sequences for the 35 high affinity CDR3 were retrieved and their frequency in the DP donor and DP-progeny data sets was determined (Supplementary Dataset 1a). For each IgE and IgG1 progeny data set, we determined the percentage of sequences present in the donor DP data set. We used a cutoff of 100 reads (cc) per sequence in the progeny data set (Supplementary Dataset 1b, c). The summary analysis showed that most high affinity IgE and IgG1 progeny sequences of spleen and bone marrow (BM) were present in the donor population (Fig. 4c). As exemplified for one high affinity CDR3 sequence all but one mutation (missense and silent) in nucleotide-encoding sequences were found in progeny and donor cells (Supplementary Table 2).

The results indicate that antigen-driven selection steers differentiation of rare DP high affinity memory clones into PC, as opposed to additional mutation and selection of memory clones.

### All IgG1 MBC subsets give rise to IgE PC in memory responses

**Fig. 5:**
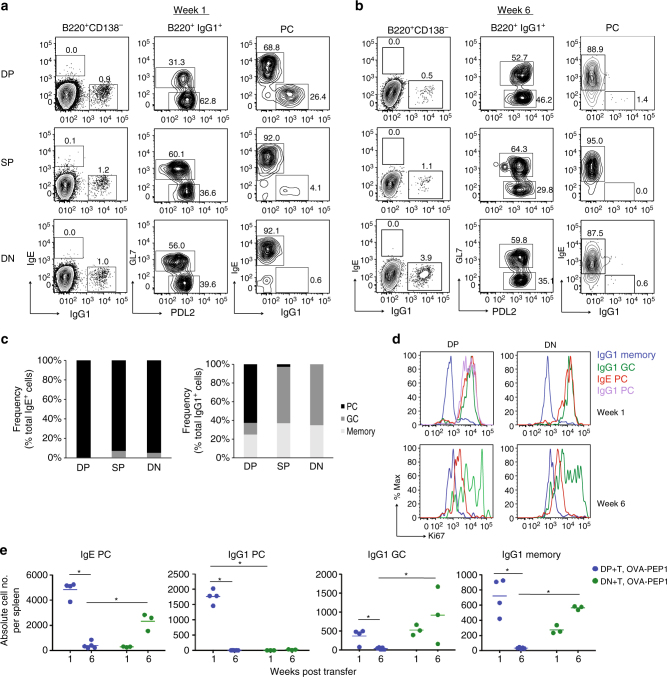
Early and late generation of IgE PC from different IgG1 MBC subsets. The phenotype of IgG1 and IgE cells generated from IgG1 MBC was analysed. IgG1 MBC subsets isolated from OVA-PEP1 immunised TBmc mice, were transferred together with CD4 memory T cells into *Rag1* KO recipients. The recipient mice were immunised with OVA-PEP1. One and 6 weeks later spleen cells were collected, stained with antibodies to B220, CD138, GL7, PDL2, IgE, IgG1 and the proliferation antigen Ki67, and analysed by flow cytometry. **a**, **b** *Left*: frequency of IgE^+^B220^+^ and IgG1^+^B220^+^ cells among total B220^+^CD138^−^ cells; *middle*: frequency of GL7^+^ GC cells and PDL2^+^ memory B cells among B220^+^CD138^−^IgG1^+^ cells; *right*: frequency of IgE^+^ PC and IgG1^+^ PC among B220^−^CD138^+^ PC. **c** Percentage of PC, GC cells and memory B cells among total IgE^+^ cells (*left*) and total IgG1^+^ cells (*right*) at 1 week after transfer/immunisation. **d** Proliferation analysis of splenic IgE^+^ and IgG1^+^ cells of recipient mice at weeks 1 and 6 after transfer/immunisation. **e** Number of IgE^+^ PC, IgG1^+^ PC, IgG1^+^ GC and IgG1^+^ MBC per spleen in recipient mice at weeks 1 and 6 after transfer/immunisation. Each *dot* represents one recipient mouse. Average of 4 (DP + T, week 1), 5 (DP + T, week 6), 3 (DN + T, week 1) and 3 (DN + T, week 6) mice per group were shown. Statistical analysis was performed using Mann–Whitney–Wilcoxon *U*-test. *P < 0.05. The data are representative of three independent experiments

To determine the fate of IgE cells derived from each IgG1 memory subset, we analysed progeny cells by flow cytometry using the TBmc OVA-PEP1 immunisation system (Fig. 2a). Briefly, IgG1 MBC subsets and CD4 memory T cells from primary immunisation were transferred to recipient *Rag1* KO mice that were then immunised with OVA-PEP1. IgG1 and IgE PC, GC and MBC in the spleens of recipient mice were quantified 1 and 6 weeks post secondary immunisation by flow cytometry analysis (Fig. 5a, b, and Supplementary Fig. 9a for gating strategy, T cell only and non-transferred controls). In all memory B and T cell recipient mice, there was a large population of endogenous B220^+^CD138^−^ B cell precursors, a small population of donor-derived B220^+^IgG1^+^ cells, and very few B220^+^IgE^+^ cells. IgE PC were derived from all IgG1 MBC subsets, but almost no IgE GC or IgE MBC were detected (Fig. 5a–c).

The majority of progeny cells retained IgG1 expression. In this case, and consistent with previous data^25^, there were clear differences between groups. Only DP cells generated a significant number of IgG1 PC cells, while both DN and SP subtypes generated mainly GC and MBC (Fig. 5a–c). GC cells and PC were Ki67^+^ proliferating cells, while most IgG1 MBC were non-proliferating Ki67^−^ cells (Fig. 5d).

Quantification of DP- and DN-derived progeny cells in the spleen of recipient mice at week 1, showed that IgE PC and IgG1 PC were most numerous in mice transferred with DP cells (Fig. 5e). In contrast, in mice that received DN cells, IgE PC were fewer in number at week 1, but increased significantly by week 6. There was a sustained generation of IgG1 GC and IgG1 MBC in mice transferred with DN IgG1 MBC.

In combination with the serum antibody kinetics (Fig. 2c, d), the cellular analysis suggests a fast but transient response by the DP memory subset, and a delayed but prolonged response by the DN subset. On day 6, donor-derived IgE and IgG1 cells were detected in recipients of DP IgG1 MBC but were still rare in recipients of DN IgG1 MBC (Supplementary Fig. 9b, c).

In summary, the fate of IgE cells derived from all IgG1 MBC subsets is restricted to PC, and thus is determined in an alternate way to the fate of the progeny that retains IgG1 expression.

### DP IgG1 MBC are poised for activation

**Fig. 6:**
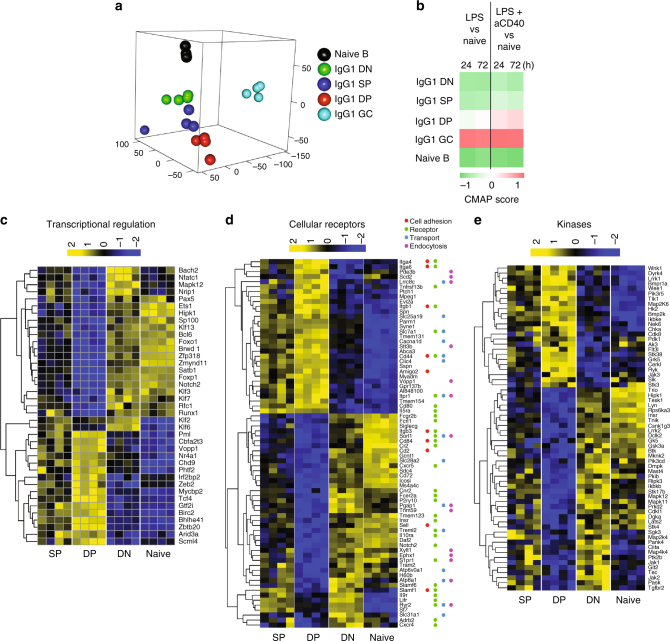
Transcriptional profile suggests readiness for activation of DP IgG1 MBC. **a** Inter-sample similarity between DP, SP and DN IgG1 MBC, IgG1 GC cells and naive B cells, visualised as a multidimensional scaling (MDS) plot. **b** Connectivity map (CMap) analysis to test the similarity of the transcriptional profile of sorted IgG1 MBC, IgG1 GC and naive B cells, with B cells activated with LPS or LPS + CD40 (GSE35998 data set). **a**–**e** The B cell populations were sorted from pooled spleen and mLN of 3 BALB/c mice at 10 weeks after infection with *N.b*. **c**–**e** Heatmaps derived from the RNA-seq analysis showed the clustering of DEG related to **c** transcriptional regulation, **d** cellular receptors and **e** kinases, among IgG1 MBC subsets (DP, SP and DN) and naive B cell samples

To explore the molecular basis of the differences between IgG1 MBC subsets, we analysed their transcriptomic profiles. IgG1 MBC subsets, IgG1 GC and naive B cells were purified in four independent experiments and analysed using RNA sequencing (RNA-seq). We identified 17,012 expressed genes in the data set. Inter-sample similarity analysis using multidimensional scaling (MDS) showed that all IgG1 MBC subsets were distantly related to IgG1 GC cells, the SP subset was intermediate between DP and DN subsets, and notably, the DN subset was the closest to naive B cells (Fig. 6a).

The functional experiments in vivo suggested that there may be pre-existing differences affecting activation among IgG1 MBC subsets. Thus, we compared their expression profiles to signatures of activated murine B cells. Differentially expressed gene (DEG) signatures of LPS, and LPS plus anti-CD40 activated B cells were generated from available data sets (GSE35998) and used in a connectivity map (CMap) analysis^31^. In the CMap, IgG1 GC cells and naive B cells yielded positive and negative scores, respectively (Fig. 6b). Strikingly, the analysis yielded positive scores for DP IgG1 MBC, and negative scores for DN and SP IgG1 MBC, indicating that DP cells were the MBC subset most related to activated cells. As MBC are non-dividing cells, these correlations may reflect their promptness to activation.

The comparison of the three IgG1 MBC subsets identified 1114 DEG (Supplementary Dataset 2a–d), of which 512 had higher expression in DP MBC, 514 in DN MBC, and only 88 in SP MBC.

Gene Ontology analysis (GO) identified transcription regulation, cell surface molecules (signalling receptors, transporters, cell adhesion molecules and endocytosis genes), and kinases as mainly represented among DEG of DP or DN subsets, with the SP subset presenting an intermediate and more heterogeneous profile (Fig. 6c–e and Supplementary Dataset 3a)^25^. Among DEG with higher expression in the DP subset were the transcription factors *Arid3a* (*Bright*) and *Gtf2i*, which regulate the *IgH* locus transcription^32^, E-box binding proteins *Tcf4*, *Bhlhe41* and *Zeb2*, and, importantly, *Zbtb20*, a regulator of PC differentiation and longevity^33, 34^. Importantly, DN IgG1 MBC expressed higher level of genes that promote the GC fate and inhibit the PC fate, such as *Bcl6*^35^, *Foxo1*^36, 37^, *Foxp1*^38, 39^, *Bach2*^40^ and *Ets1*^38^. Metacore analysis of pathway/processes indicate B cell receptor (BCR) pathway, cell adhesion, proliferation, cytokine signalling, among the top enriched entities (Supplementary Dataset 3b, c). In the BCR pathway it is worth pointing out the upregulation, in the DP subset, of the IP_3_ receptor gene *Itpr3*, and of *Pdk1* and *Lrrk1*, supporting promptness to BCR mediated calcium release and NF-κB activation.

A microarray analysis of memory B cell subsets was published by Zuccarino-Catania et al.^25^. The expression of DEG identified by Zuccarino-Catania et al. was determined in our RNA-seq data set. The comparison demonstrates many common DEG in DP and DN subsets analysed in both studies (Supplementary Fig. 10a). Among common DEG are genes known to impact PC and GC cell fate, such as *Zbtb20* (PC) and *Bcl6* (GC). There are however some differences between that expression analysis and ours presented herein. For microarray analysis, Zuccarino-Catania et al. used total memory B cells (thus containing all isotypes), sorted based on PDL2 and CD80 expression^25^, while we sorted PDL2^+^IgG1^+^ memory B cells based on differential expression of CD73 and CD80. CMap comparison of both data sets demonstrated similarity between DP populations but not SP or DN populations (Supplementary Fig. 10b). This is explained by the fact that DP subsets in both studies expressed PDL2, CD80 and CD73, and that the DP subset in the microarray study was enriched in IgG1 cells, as shown by the differential expression of *Ighg1* (Supplementary Fig. 9b).

Overall, the functional analysis and RNA-seq expression data of IgG1 MBC subsets in our work indicate that the DP IgG1 MBC are mainly ‘pro-PC’ cells, while the DN IgG1 MBC are ‘pro-GC’ cells. Nevertheless, IgE daughter cells derived from either DP or DN IgG1 MBC were PC. Thus, IgE expression overrides the precursor MBC differentiation bias, determining a dominant PC fate.

### Pathogenicity of IgE derived from DP and DN IgG1 MBC

**Fig. 7:**
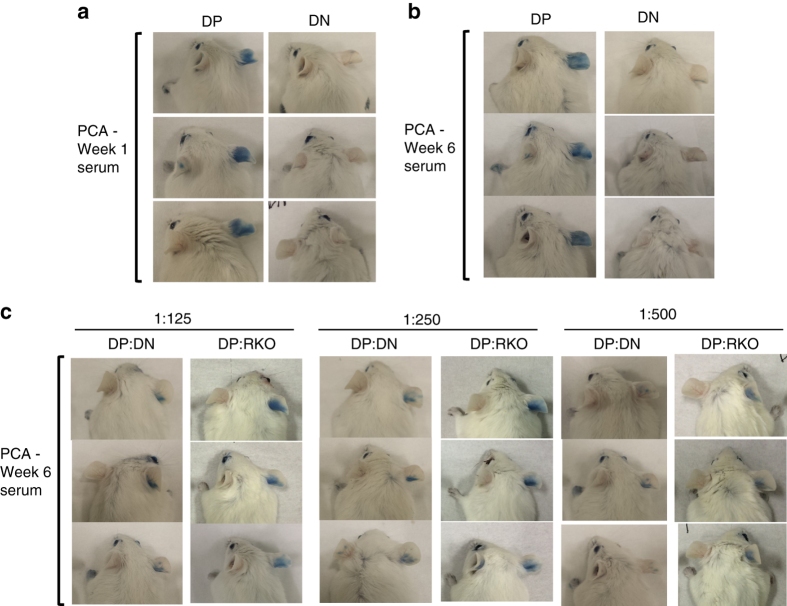
IgE derived from DP MBC mediates anaphylaxis. The pathogenic capacity of IgE derived from DP and DN IgG1 MBC was evaluated in a passive cutaneous anaphylaxis (PCA) assay. IgG-depleted serum was obtained from *Rag1* KO recipient mice that had been transfused with DP or DN IgG1^+^ MBC and CD4 memory T cells from primary OVA-PEP1 immunised TBmc mice as described in Fig. 2a, c, d. The recipient mice were immunised with OVA-PEP1, and serum was obtained at 1 and 6 weeks after transfer/immunisation. Serum from each group was pooled and depleted of IgG antibodies. For the PCA assay, 20 μl of serum was injected intradermally into the ears of naive BALB/c mice. Twenty-four hours later, 50 µg OVA-PEP1 and 1% Evans Blue in PBS were injected intravenously. Thirty minutes later, the anaphylactic reaction was evaluated visually. **a**, **b** Sera from recipients of DP IgG1 MBC (DP-serum), but not from recipients of DN IgG1 MBC (DN-serum), mediated anaphylaxis. **a** One-week sera. **b** Six-week sera. **c** To determine if the DN-serum could inhibit anaphylaxis, 6-week DP-serum was diluted with 6-week DN-serum, or with serum from untreated *Rag1* KO mice (RKO serum) at the indicated DP:DN and DP:RKO ratios (1:125; 1:250; 1:500). The mixed serum anaphylactic activity was measured in a PCA assay. The data are representative of two experiments

Having established different roles for the IgG1 MBC subsets in IgE memory responses, we assessed the pathogenic potential of IgE derived from DP and DN IgG1 MBC using the passive cutaneous anaphylaxis (PCA) model. We used TBmc mice immunised with OVA-PEP1 as a source of donor IgG1 MBC and an adoptive transfer system as described above (Fig. 2a–d). We obtained serum from recipient mice transferred with either DP or DN IgG1 MBC (referred as DP- and DN-serum, respectively) at 1 and 6 weeks after immunisation. Twenty microlitres of IgG-depleted DP-serum and DN-serum were injected into the ears of naive BALB/c mice (Fig. 7a, b). Twenty-four hours later, the mice were injected intravenously with OVA-PEP1 and 1% Evans Blue and the reactions were evaluated thirty minutes later.

DP-serum from weeks 1 and 6 mediated anaphylaxis, while DN-serum did not (Fig. 7a, b). When 6-week DN-serum was added to pathogenic 6-week DP-serum at DP:DN ratio of 1:125 and 1:250, the DN-serum did not increase the PCA reactions but appeared to have an inhibitory activity when compared to control *Rag1* KO (RKO) serum added to DP-serum (Fig. 7c). At a 1:500 ratio, dilution of the DP-serum accounted for decreased PCA, as the PCA intensity was similar between DP:DN and DP:RKO injected mice (Fig. 7c). No inhibition was observed at DP:DN ratios of 1:60 (Supplementary Fig. 11a) suggesting that at least a 100-fold or higher excess of low affinity/non-specific IgE to high affinity IgE may be needed to prevent mast cell degranulation. Although 6-week DN-serum did not mediate anaphylaxis to the immunising antigen PEP1 due to low binding, it had the ability to mediate anaphylaxis if crosslinked (Supplementary Fig. 11b).

The results demonstrate that IgE derived from DP IgG1 MBC is of high affinity and pathogenic, while IgE derived from DN IgG1 MBC is of low affinity and does not contribute to anaphylaxis.

## Discussion

The mechanisms that generate and maintain the memory of IgE responses are poorly understood. In previous work, we showed that the differentiation of IgE cells follows a unique pathway^4, 10^ characterised by a transient GC phase, a predominance of the PC phenotype, and the lack of bona fide IgE MBC^2, 3, 6^. This suggested that non-conventional mechanisms are involved in the memory of IgE responses. Previous work from our laboratory using IgG1-deficient mice, demonstrated that IgG1 cells were essential to generate high affinity IgE in repeatedly immunised mice^5^. Since IgG1-deficient mice still produced low affinity IgE derived from direct switching of IgM cells, the results indicated that neither naive nor memory IgM cells were involved in generating high affinity IgE. Given the prominent role of sequential switching of IgG1 cells in the generation of high affinity IgE^5^, we hypothesised that IgG1 MBC were the precursors of IgE cells in memory responses. Using previously described MBC markers^23, 25^, we identified three main IgG1 MBC subsets, CD73^+^CD80^+^ (DP), CD73^−^CD80^+^ (SP) and CD73^−^CD80^−^ (DN) cells, and tested their ability to give rise to IgE-producing cells in a recall response. We found that only the DP IgG1 MBC subset generated IgE PC producing high affinity IgE capable of mediating anaphylaxis. SP and DN IgG1 MBC also gave rise to IgE PC, however, their response were delayed and the IgE antibodies produced were of low affinity and non-pathogenic.

In contrast, IgG1 PC was mainly generated from the DP IgG1 MBC and contained predominantly high affinity clones. Interestingly, within the progeny of the same DP IgG1 MBC, more IgG1 than IgE cells carried high affinity BCR mutations. The conditions that drive the same DP IgG1 MBC to generate IgG1 or IgE PC remain a major unresolved issue.

Our observations predict that the IgE antibody pool would tend to be more heterogeneous and of lower average affinity than the IgG1 antibody pool. In fact, we observed delayed affinity maturation of the IgE response compared with the IgG1 response over the course of repeated immunisations in mice^6^. Low affinity/allergen non-specific IgE, and high affinity IgG1, may protect from allergic reactions driven by high affinity IgE in different ways. Low affinity/allergen non-specific IgE bound to mast cells would dilute high affinity IgE on the surface of mast cells, decreasing the probability of mast cell degranulation. High affinity IgG1 may neutralise allergens in circulation, reducing their availability. In addition, allergen-IgG1 immune complexes bound to FcγRIIB on mast cells would negatively regulate FcεRI signalling^41^.

When comparing the frequency of high affinity clones between the DP IgG1 MBC and their IgE and IgG1 PC progeny, we observed a striking enrichment of high affinity clones in the PC progeny. We asked if this occurred through direct expansion of pre-existing memory B cell clones, or if it required further somatic mutation and selection. By comparing the nucleotide sequences encoding high affinity CDR3 H domains of donor and progeny B cells, including silent mutations with no selective value, we discovered that most high affinity CD3-encoding nucleotide sequences in progeny cells were present in the donor memory B cells. These findings support a model whereby high affinity PC are derived from memory cell clones by selection and expansion without further mutagenesis. It is thus likely that clonal selection of IgG1 MBC would require specific antigen capture through high affinity BCR, and antigen processing and presentation to antigen-specific CD4 T cells. Analogous to GC cells that become PC^42^, high affinity DP IgG1 MBC clones appear to have the greatest ability to become PC.

Generation of IgE PC from DP IgG1 MBC implies that the enzyme activation-induced cytidine deaminase (AID), which mediates class-switch recombination and somatic hypermutation^43^, is expressed and functions in at least a subset of activated DP IgG1 MBC. Since most high affinity PC derived from the DP IgG1 MBC subset do not carry additional mutations, and differentiation of IgG1 MBC into PC is fast, an intermediate GC phase before PC differentiation appears unlikely.

Interestingly, there was very little or no selection of high affinity memory clones in the differentiation of DN IgG1 MBC to IgE PC. This was surprising because the DN IgG1 MBC donor pool did contain high affinity clones, albeit at a lower frequency than the DP IgG1 MBC. Since DN IgG1 MBC differentiate into GC cells, and T-B cell interactions are essential for GC formation and T follicular helper cell differentiation^44–46^, the DN response must depend on interactions with T helper cells. It is possible that DN IgG1 MBC with low affinity BCR are also preferentially selected for GC differentiation. This would ensure that the new memory pool, which is mostly derived from the DN IgG1 MBC (this article and Zuccarino-Catania et al.^25^), is of a broad affinity range. DN IgG1 MBC that switch to IgE may be also co-opted from low affinity clones and prevented to become GC cells by the intrinsic PC-driving activity of membrane IgE^47^.

Interestingly, we found that the expression profile of DP IgG1 MBC was closer to that of activated B cells than to naive B cells, while the reverse was true for DN IgG1 MBC. DP IgG1 MBC appeared poised for rapid activation and differentiation into PC, expressing higher levels of transcription factors that regulate the IgH locus transcription, as well as *Zbtb20*, a regulator of PC survival and longevity^33^. On the other hand, DN IgG1 MBC expressed higher levels of several inhibitors of activation, and of genes that inhibit PC differentiation and promote the GC fate, such as *Bcl6*^35^, *Foxp1*^38, 39^ and *Bach2*^40^.

Interestingly, we described that IgE GC cells did not have switch footprints from the Sγ1 regions, and thus were derived from IgM cells and not from IgG1 cells^3^. This is entirely consistent with the lack of IgE GC cells in the secondary response of IgG1 MBC shown in this manuscript.

In summary, our studies revealed an important role of IgG1 MBC subsets in generating and maintaining the memory of IgE responses. DP IgG1 (CD73^+^CD80^+^) MBC provided fast production of high affinity IgE antibodies, while the DN IgG1 (CD73^–^CD80^−^) MBC gave rise to a delayed response of low affinity non-pathogenic IgE. In addition, we showed that IgG1 MBC subsets have alternate functions with regards to the production of IgE and IgG1 subsets. Activation of IgG1 MBC results in rapid production of high affinity IgG1, formation of new IgG1 GC cells, and expansion of the IgG1 memory pool. In contrast, IgE cells generated from IgG1 MBC are exclusively IgE PC that secrete antibodies of broader affinity than IgG1 antibodies. These mechanisms restrict the production of high affinity IgE and reduce the pathogenicity of the IgE pool.

Our repertoire analysis supports a model whereby rapid production of high affinity antibodies in memory responses is an antigen-driven selection process that does not involve further mutagenesis. The results presented here demonstrate a fundamental but unexpected facet of the control of IgE pathogenesis, and provide insights into the mechanisms of antibody production in memory responses. These findings have implications for the understanding of humoral immunity to infections and vaccinations.

## Methods

### Mice immunisations and infections

BALB/c (stock 000651), 4get BALB/c^28^ (stock 004190) and *Rag1* KO BALB/c (stock 003145) mice were purchased from The Jackson Laboratory. CB17 mice (BALB/c congenic for the *IgH*^*b*^ allele, stock C.B-Igh1b/lcrTac) were purchased from Taconic Biosciences. TBmc mice^6, 26^ were crossed with 4get mice to generate 4get TBmc mice. All mice were housed in the specific pathogen-free animal facility of the Biological Research Center (BRC) A*STAR, Singapore. Male and females mice between 6 and 8 week of age were used for experiments. Mice were killed by CO_2_ inhalation. All animal procedures were approved by the BRC/A*STAR and NYU School of Medicine Institutional Animal Care and User Committee. TBmc mice and 4get TBmc mice were immunised by intraperitoneal injection of 100 µg OVA-PEP1 (chicken ovalbumin (OVA), Sigma catalogue # A2512, conjugated to peptide YPYDVPDFASLRS (PEP1)) in alum^6^. BALB/c and 4get BALB/c mice were infected subcutaneously with 500 L3 larvae of *N.b*.

### Flow cytometry analysis and cell sorting

Single-cell suspensions were incubated with 10 µg/ml anti-CD16/32 (eBioscience, clone 93) for 15 min at 4 °C before surface labelling with antibody cocktails in staining buffer (2% FBS, 4 mM EDTA and 0.1% NaN_3_) for 30 min at 4 °C. Intracellular staining of Ki67 was conducted using anti-Ki67 antibody (clone 16 A8) and Foxp3 staining buffer as described by the manufacturer (eBioscience, catalogue # 00-5521). Cell surface IgE staining was performed using R1E4 antibody^48^ which does not recognise FcεRI-bound or FcεRII- bound IgE^3^. Cells were analysed using an LSR II 5-laser flow cytometer (BD) or sorted using a FACSAria II 5-laser cell sorter (BD). Purified cells were used for transcriptomic analysis, VDJ repertoire sequencing, or for adoptive transfer experiments. Memory B cells (MBC) were isolated from pooled spleen and mesenteric LN (mLN) of TBmc mice at 10 weeks after immunisation with OVA-PEP1 in alum, or from BALB/c mice 10 weeks after infection with *N.b*. Memory B cells were pre-enriched by magnetic negative selection sorting using biotinilated antibodies to CD3e (clone145-2C11), IgD (clone 11-26c), CD138 (clone 281-2) and TER-119 (clone TER-119), and streptavidin-labelled magnetic beads (Miltenyi Biotec, catalogue # 130-090-485). Negatively selected cells were stained with antibodies eVolve605 anti-CD4 (clone RM4-5), eFluro450 anti-IgM (clone II/41), APC-eFluro780 anti-B220 (clone RA3-6B2), PE-Cy7 anti-IgG1 (clone M1-14D12), PE anti-CD80 (clone 16-10A1), PerCP-eFluro710 anti-CD73 (TY/11.8), from eBioscience, and BUV395 anti-PDL2 (clone TY25) and APC anti-GL7 (clone GL7), from BD Bioscience. For flow cytometry sorting, the cells were first gated as IgM^−^CD4^−^IgG1^+^B220^+^GL7^−^PDL2^+^ (IgG1^+^ memory B cell gate), and the CD73^+^CD80^+^ (DP), CD73^−^CD80^+^ (SP) and CD73^−^CD80^−^ (DN) subsets were subsequently sorted. T cells were isolated from pooled spleen and mLN of 4get TBmc mice at 10 weeks after immunisation with OVA-PEP1, or from 4get BALB/c mice at 10 weeks after infection with *N.b*. Single-cell suspensions from the pooled spleen and mLN were pre-enriched for CD4^+^ T cells using the Miltenyi T cell isolation kit II (Miltenyi Biotec, catalogue # 130-095^−^130). The pre-enriched cells were then stained with the following antibodies: Alexa700 anti-CD4 (clone GK1.5), APC anti-CD45RB (clone C363.16A) and PerCP anti-CD25 (clone PC61.5), from eBioscience. CD4^+^CD25^–^CD45RB^low/int^GFP^+^ cells were sorted by flow cytometry and are referred to as CD4 memory T cells. A list of all antibodies used is provided in Supplementary Table 3.

### Adoptive transfer experiments

IgG1 MBC subsets and GFP^+^CD4^+^ CD4 memory T cells were isolated from mice at 10 weeks after primary immunisation or infection. CD4 memory T cells were obtained from 4get mice. Sorted 10^5^ MBC together with 5 × 10^4^ CD4 memory T cells were injected intravenously into *Rag1* KO mice, or low dose (150 rad) γ-irradiated BALB/c or CB17 mice. For irradiation a Gammacell 40 Exactor irradiator with a ^137^Cs source was used. The recipient mice were subsequently immunised or infected, matching the treatment of the donor mice. Spleen, BM and serum were collected at indicated time points for analysis of serum antibodies, flow cytometry analysis of donor-derived B cells, or for collecting of RNA.

### Serum antibodies

Serum levels of PEP1- and HA- specific IgE and IgG1, total IgE and IgG1, or total IgE^a^ and IgG1^a^ antibodies (in CB17 recipients), were determined by ELISA as described^6^. To measure antigen-specific IgE, the sera were first depleted of IgG antibodies by incubation with GammaBind Plus Sepharose (GE Healthcare, catalogue # 17088601). For antigen-specific ELISA assays, 96-well plates were coated with 2 μg/ml HA (YPYDVPDYASLRS) or PEP1 peptides in PBS. For total IgE and IgG1 assays, plates were coated with 2 μg/ml of rat-anti-mouse IgE (clone LO-ME-3, Invitrogen) or goat F(ab’)_2_ anti-mouse IgG1 (Southern Biotech) respectively. The plates were incubated overnight at 4 °C, washed with washing buffer (PBS + 0.05%Tween-20), and blocked with blocking buffer (PBS + 0.05%Tween-20 + 1% bovine serum albumin), for 1 h at room temperature. Serial dilution of serum samples and standard isotypes (IgE: BD Bioscience, clone IgE-3; IgG1: BD Bioscience, clone MOPC-21) in blocking buffer were then added to wells in duplicate and the plates were incubated 4 h at room temperature and then washed. For detection of IgE antibodies, the plates were incubated for 1 h at room temperature with goat anti-mouse IgE-HRP (Southern Biotech). For IgE^a^ detection, the plates were first incubated with anti-mouse IgE^a^-biotin antibody (clone UH297, BioLegend), followed by streptavidin HRP (BD Pharmingen, catalogue # 554066). For IgG1 detection, goat anti-mouse IgG1-biotin antibody (Southern Biotech), followed by incubation with streptavidin HRP. For IgG1^a^ detection, the plates were incubated with anti-mouse IgG1^a^-biotin (clone 10.9, BD Bioscience) was added to each well, followed by incubation with Streptavidin HRP. After washing, 100 µl TMB substrate solution (eBioscience, catalogue # 00-4201-56) was added to the wells. The reaction was stopped by addition of 50 µl 2 N H_2_SO_4,_ and optical density was then measured at 450 nm using Tecan Plate Reader (reference at 590 nm).

### Passive cutaneous anaphylaxis assay

For the passive cutaneous anaphylaxis assay (PCA), 20 µl of IgG-depleted serum was injected intradermally into the ears of naive BALB/c mice. Twenty-four hours later, the mice were injected intravenously with 50 μg of OVA-PEP1 and 1% Evans Blue in PBS and were sacrificed 30 min later. The extravasation of Evans Blue, indicating increased vascular permeability due to anaphylaxis, was recorded by photography.

### IgE and IgG1 VDJ H repertoire analysis

TBmc and 4get TBmc mice were immunised with OVA-PEP1 in alum. DP, SP and DN IgG1^+^ MBC were isolated from pooled spleen and mLN of TBmc mice 10 weeks after immunisation. A fraction of each MBC purified subset was used for RNA extraction. Purified live IgG1^+^ MBC were injected intravenously into *Rag1* KO mice together with CD4 memory T cells isolated from immunised 4get TBmc mice. Recipient mice were immunised with OVA-PEP1 in alum. Two weeks later, spleen and BM were collected for RNA extraction.

Total RNA from donor IgG1^+^ MBC and from spleen and BM of recipient mice were extracted using RNeasy Mini Kit (QIAGEN, catalogue # 74106). cDNA was then synthesised using SuperScript II Reverse Transcriptase (Thermo Fisher, catalogue # 18064014), and IgE and IgG1 amplicon sequence libraries were prepared by two-rounds of PCR amplification using LongAmp *Taq* DNA polymerase (New England Biolabs, Inc., catalogue # M0323L). The reaction mix for the first round of 15-cycles amplification contained a forward primer (5′-TGAAACTCTCCTGTGCAGCC) binding to the V region and reverse primers specific for the first exon of Cε (5′-AAGGGGTAGAGCTGAGGGTTC-3′) or Cγ1 (5′-TAGACAGATGGGGGTGTCGT-3′). For the second round of PCR amplification, barcodes for identifying and de-multiplexing individual samples, and Illumina adapter sequences were added to the template. Amplification was carried out for 30 cycles. Amplicons in the size range of 450–500 base pairs (bp) were gel purified using Qiaquick Gel Extraction Kit (Qiagen, catalogue # 28704) and quantified using Quant-iT Picogreen dsDNA Assay kit (Invitrogen, catalogue # P11496). qPCR was performed on the amplicon library to ascertain the loading concentration using KAPA library quantification kit (KAPA Biosystems, Catalogue # KK4854). The library was sequenced using the Illumina MiSeq to generate 250 bp paired-end reads at a sequencing ranging from 0.1 to 0.8 million reads per sample.

The VDJ sequences were analysed with the MiXCR software^49^. Clonotypes were clustered based either on CDR3 alone or CDR1, CDR2 and CDR3 together. Resulting clonotypes were then further grouped by amino acid identity. Selected clonotypes were further characterised by DNA identity. Sequence alignment, clustering and general data morphing was performed with custom scripts in pipeline pilot (www.accelrys.com). The VDJ H data sets have been deposited in NCBI Bioproject, under the accession code PRJNA319596.

### Quantitative PCR analysis of immunoglobulin transcripts

Total RNA from spleen cells was isolated using TRIzol (Thermo Fisher, catalogue # 15596026). Total RNA from sorted naive B cells and IgG1^+^ MBC were isolated using the Arcturus PicoPure RNA Isolation Kit (Thermo Fisher, catalogue # KIT0204). cDNA was synthesised using SuperScript II Reverse Transcriptase (Thermo Fisher, catalogue # 18064014). Quantitative PCR (QPCR) for selected gene transcripts was carried out on an Applied Biosystems StepOnePlus Real-Time PCR system using Power SYBR Green PCR Master Mix (Thermo Fisher Scientific, #4367659). The following oligonucleotide pairs were used: 5′-GTACGACGAGAACGGGTTTG-3′ and 5′-AGTTCACAGTGCTCATGTTCAG-3′ for Cε mature transcript; 5′-GTACGACGAGAACGGGTTTG-3′ and 5′-GGATCCAGAGTTCCAGGTCACT-3′ for Cγ1 mature transcript; 5′-TGACAGGATGCAGAAGGAGA-3′ and 5′-GTACTTGCGCTCAGGAGGA-3′ for *β-Actin* trancript. Gene expression of mature Cε and Cγ1 transcripts was normalised to *β-Actin*.

### Transcriptomic analysis

RNA was isolated from sorted B cell subsets of BALB/c mice 10 weeks after infection with *N*.*b*. using Arcturus PicoPure RNA Isolation Kit (Life Technologies, catalogue # KIT0214). RNA quality was assessed by a Bioanalyser (Agilent Technologies), all samples had RIN ≥ 7.4. From each sample, 2 ng of RNA were subjected to cDNA synthesis using NuGEN Ovation RNA-Seq System V2 kit (Integrated Sciences, catalogue # 7102-A01-NUG) according to the manufacturer’s protocol. Libraries were made using NuGENEncore Rapid Library Systems kit (Integrated Sciences, catalogue # 0347-A01-NUG) according to the manufacturer’s protocol with 500 ng of cDNA. The libraries were subjected to an indexed paired-end sequencing run of 2 × 51 bp on an Illumina HiSeq 2000 sequencer.

The RNA sequencing reads were subjected to quality control testing using FastQC (ver. 0.10.0, http://www.bioinformatics.babraham.ac.uk/projects/fastqc/). Quality checked, paired-end reads were aligned to the mm9 reference genome assembly using STAR aligner^50^. Reads were allowed to map across splicing junctions as per gencode mouse version M1 annotations^51^. FeatureCounts^52^ was used to calculate read counts covering each gencode gene. Then edgeR package^53^ was used to normalise raw read counts over genes to counts per million mapped reads (CPM) using the TMM method. Low abundance genes with CPM < 1 in all cell types were filtered out and subsequently differential gene expression analysis was performed in edgeR with a negative binomial model. DEG across cell types were defined based on a 0.05 false discovery rate (FDR) and a minimum expression threshold of log_2_CPM > 1. Enrichment analysis of gene ontology terms in DEG was performed using DAVID version 6.8^54^. Signalling pathways and biological process networks significantly modulated by the DEG were identified using Metacore software suite licensed from Thomson Reuters Life Sciences Research. Connectivity map (CMap) analysis comparing RNA-seq gene expression profiles with gene expression signatures derived from public data was performed using the ‘gCMap’ package in R Bioconductor (version 1.16.0). The expression data sets have been deposited in NCBI GEO under the accession code GSE83436.

### Statistics

The *P* value calculations did not assume equal variance across groups. Data in Figs 2b, 5e were compared with non-parametric Kruskal–Wallis test for multiple comparisons followed by Mann–Whitney–Wilcoxon *U*-test (two-tailed) for paired groups. Non-parametric Kruskal–Wallis rank-sum test was used to calculate *P* value in Fig. 2c, d and Supplementary Fig. 2a.

### Data availability

The repertoire data sets generated and analysed during the current study are available in NCBI Bioproject, under the accession code PRJNA319596. The transcriptomic data sets are available in NCBI GEO under the accession code GSE83436.

## Electronic supplementary material

Supplementary Information

Description of Additional Supplementary Files

Supplementary Dataset 1

Supplementary Dataset 2

Supplementary Dataset 3
