# Publisher Correction: IgG1 memory B cells keep the memory of IgE responses

**DOI:** 10.1038/s41467-018-03364-z

**Published:** 2018-03-01

**Authors:** Jin-Shu He, Sharrada Subramaniam, Vipin Narang, Kandhadayar Srinivasan, Sean P. Saunders, Daniel Carbajo, Tsao Wen-Shan, Nur Hidayah Hamadee, Josephine Lum, Andrea Lee, Jinmiao Chen, Michael Poidinger, Francesca Zolezzi, Juan J. Lafaille, Maria A. Curotto de Lafaille

**Affiliations:** 1 Singapore Immunology Network (SIgN), 8A Biomedical Grove, Singapore, 138648 Singapore; 2 School of Biological Sciences, Nanyang Technological University, 60 Nanyang Drive, Singapore, 637551 Singapore; 3 Division of Pulmonary, Critical Care and Sleep Medicine, Departments of Medicine and Cell Biology, New York University School of Medicine, 550 First Ave, NY, 10016 USA; 4 Galderma R&D, Les Templiers, 2400 route des Colles, Sophia Antipolis, 06410 Biot France; 5 Skirball Institute and Department of Pathology, New York University School of Medicine, 540 First Ave, NY, 10016 USA

Correction to: *Nature Communications* 10.1038/s41467-017-00723-0, published online 21 September 2017

The originally published version of this Article contained errors in Fig. 4 that were introduced during the production process. In panel c, the two uppermost labels ‘IgE spleen’ and ‘IgE BM’ incorrectly read ‘IgG1 spleen’ and ‘IgE1 BM’, respectively. These errors have now been corrected in both the PDF and HTML versions of the Article.

