# Supplementary material for: IgG1 memory B cells keep the memory of IgE responses
Source: Nat Commun. 2017 Sep 21;8:641. doi: 10.1038/s41467-017-00723-0 (PMC5608722; doi:10.1038/s41467-017-00723-0)
Supplement: Supplementary file 1 — Supplementary Information [file 41467_2017_723_MOESM1_ESM.pdf]

Supplementary Figure 1

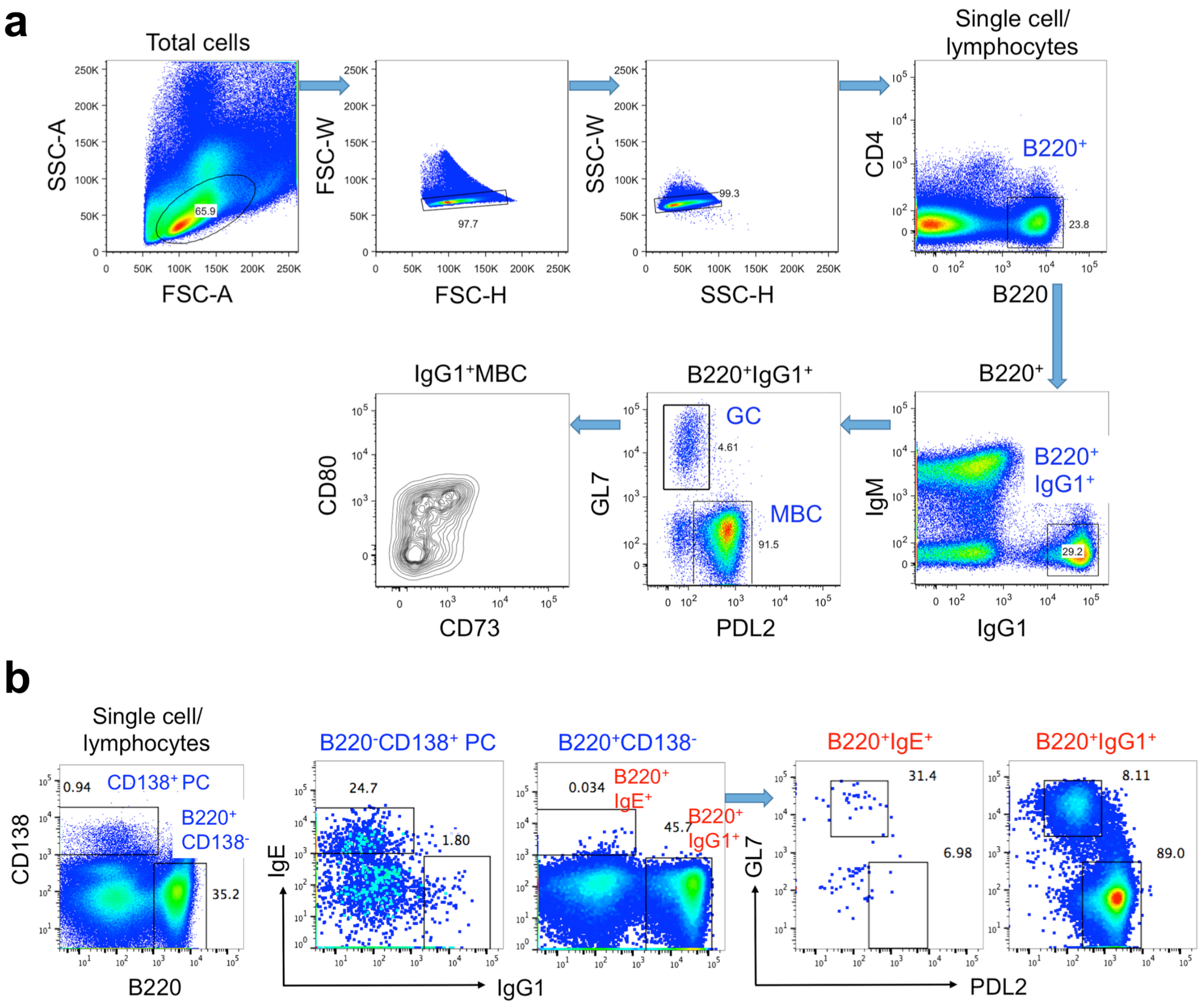

Supplementary Figure 1. Analysis of IgE and IgG1 B cells in immunised and infected mice. TBmc mice were immunised with OVA-PEP1 in alum by intraperitoneal route. BALB/c mice were infected with *Nippostrongylus brasiliensis* by subcutaneous route. Ten weeks later, spleen and mesenteric LN were harvested for flow cytometry analysis. Cells were pre-enriched by magnetic-beads depletion of CD3e$^+$, IgD$^+$, CD138$^+$ and TER-119$^+$ cells. Enriched cells were then stained with antibodies to CD4, IgM, B220, IgG1, CD80, CD73, PDL2 and GL7, and were analysed by flow cytometry. (**a**) The gating strategy to identify IgG1$^+$ germinal centre (GC) cells and memory B cells (MBC). A lymphocyte gate was first defined by SSC-A/FSC-A fluorescence, and doublets were excluded by sequential FSC-W/FSC-H and SSC-W/SSC-H gating. Consecutive gating of B220$^+$, B220$^+$IgG1$^+$, and B220$^+$IgG1$^+$PDL2$^+$ cells identified the IgG1 MBC. IgG1 GC cells were identified by GL7 expression. The same flow cytometry analysis strategy was used to generate the data in **Fig.1 a-b**. (**b**) The presence of IgE cells in 10-weeks immunised TBmc mice was determined using flow cytometry. A representative analysis is shown. Spleen and mesenteric LN cells were depleted of CD3e$^+$, IgD$^+$, and TER-119$^+$ cells by magnetic sorting, were then stained with antibodies to B220, CD138, IgG1, IgE, PDL2 and GL7, and were analysed by flow cytometry. CD138$^+$B220^{low} plasma cells (PC) and CD138$^-$B220$^+$ B cells (left plot) were identified in a single cell/lymphocyte gate defined by FSC/SSC pattern as above. The second and third plots from the left show IgE and IgG1 cells in the PC gate (second plot) and the CD138$^-$B220$^+$ B cell gate (third plot). The presence of GC and MBC among gated B220$^+$IgE$^+$ and B220$^+$IgG1$^+$ cells were determined by expression of GL7 and PDL2 respectively (two right plots). Very few PDL2$^+$IgE$^+$ cells were found in the IgE$^+$ gate (6 events in 732706 of lymphocyte gate, or 0.0008% of lymphocytes), while a robust PDL2$^+$IgG1$^+$ memory population was observed (103585 in 732706 of lymphocyte gate, or 14% of lymphocytes). In contrast to the paucity of IgE$^+$ memory B cells, there was a sizable population of CD138$^+$IgE$^+$ PC (1674 cells in 732706 or 0.22% of lymphocytes).

Supplementary Figure 2

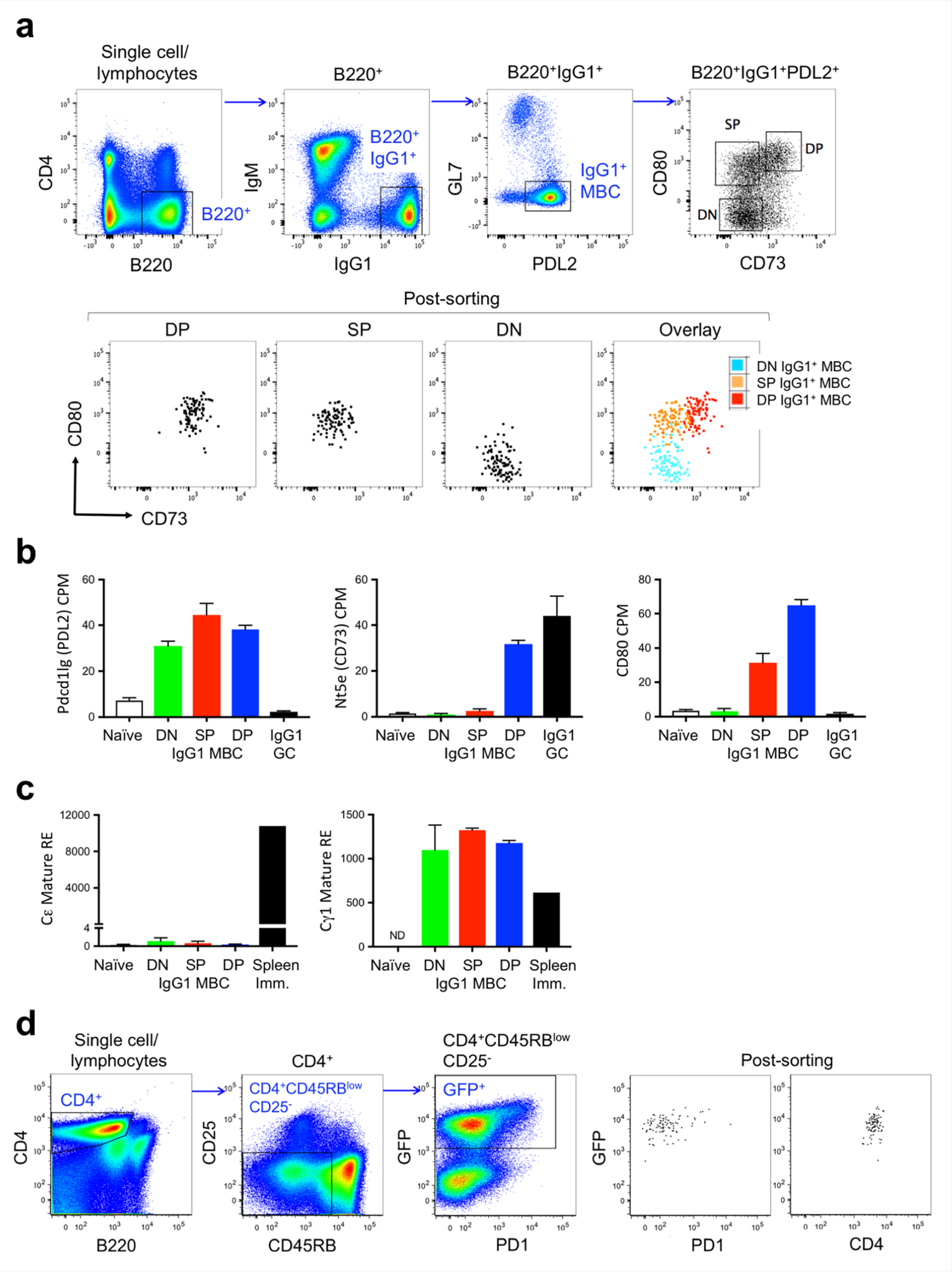

Supplementary Figure 2. Purification of IgG1$^+$ memory B cells and CD4$^+$ memory T cells. Single cell suspensions of spleen and mesenteric LN cells were prepared from OVA-PEP1 immunised TBmc mice and from OVA-PEP1 immunised TBmc 4get mice, and the cells were processed for purification of IgG1 MBC subsets and IL-4-producing memory T CD4 cells respectively, as described in the Methods section. **(a)** Gating strategy for flow cytometry sorting of double positive (DP), single positive (SP) and double negative (DN) IgG1$^+$ MBC subsets based on CD73 and CD80 expression (upper row), and post sorting analysis of the purified IgG1$^+$ MBC subsets (lower row). (**b**) Transcriptional expression of PDL2, CD80 and CD73 in purified IgG1 MBC subsets (obtained from the RNAseq dataset, n=4) corroborated the subset identity. (**c**) Quantitative real time PCR analysis of mature Cε transcript (Cε Mature) and Cγ1 transcript (Cγ1 Mature), in sorted naïve B cells (n=2) and IgG1$^+$ MBC subsets (DP, SP and DN; n=3), and in total spleen of immunised TBmc mice (containing approximately 0.1% of IgE$^+$ PC). RE: relative expression. The analysis demonstrated lack of contaminant IgE cells in the purified IgG1$^+$ MBC subsets. **(d)** Gating strategy for flow cytometry sorting of IL-4-producing GFP$^+$CD4$^+$ memory T cells and post-sorting analysis. (**a,d**) Single cell/lymphocyte gates were defined by FSC/SSC pattern as in **Supplementary Fig.1a**.

Supplementary Figure 3

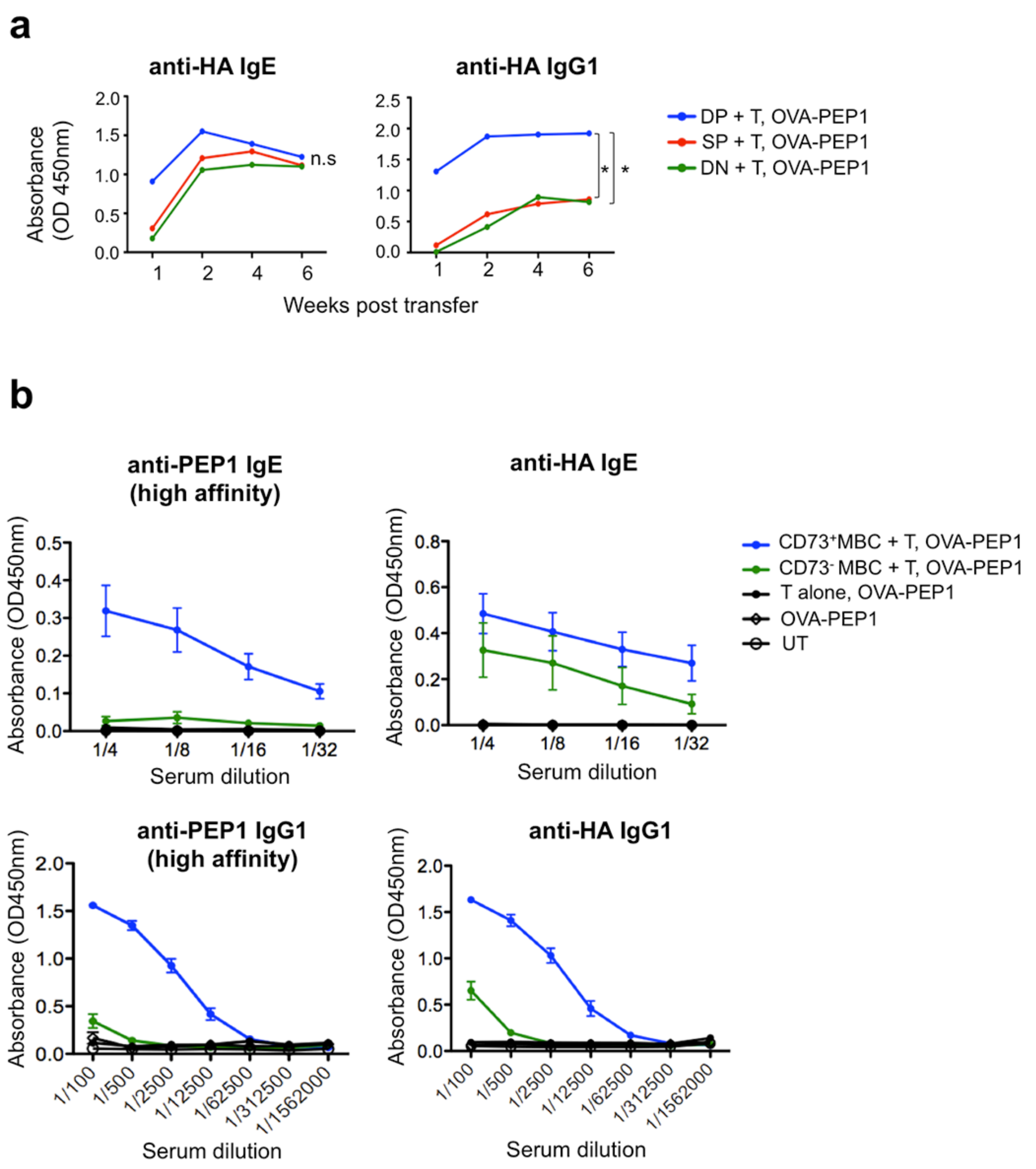

Supplementary Figure 3. CD73$^+$ and CD73$^-$ IgG1 MBC give rise to IgE PC. (**a**) Kinetics of the production of HA specific IgE and IgG1 in serum of *Rag1* KO mice that were transferred with DP, SP or SP IgG1 MBC and CD4 memory T cells from OVA-PEP1 immunised TBmc mice, and were subsequently immunised with OVA-PEP1. Binding to the cognate B cell antigen HA detects non-mutated antibodies. The data for each time point was obtained with pooled serum from 4 (DP and SP) and 3 (DN) recipient mice per group. The serum was diluted 500 fold to measure HA-specific IgE and IgG1. UT: untreated. Non-parametric Kruskal-Wallis rank sum test was used to calculate the *P* value. *: $P < 0.05$; n.s.: not significant ($P > 0.05$). (**b**) IgG1$^+$PDL2$^+$CD73$^+$ and IgG1$^+$PDL2$^+$CD73$^-$ MBC were isolated from OVA-PEP1 immunised mice at 10 weeks post-immunisation, and transferred together with CD4 memory T cells into BALB/c mice. The recipient mice were immunised with OVA-PEP1. Serum was collected 2 weeks after transfer/immunisation. High affinity IgE and IgG1 antibodies to the immunising B cell antigen PEP1, and IgE and IgG1 antibodies to the B cell cognate antigen HA were measured by ELISA. The results are shown as mean ± SEM of 3-4 mice per group. Data are representative of two independent experiments.

Supplementary Figure 4

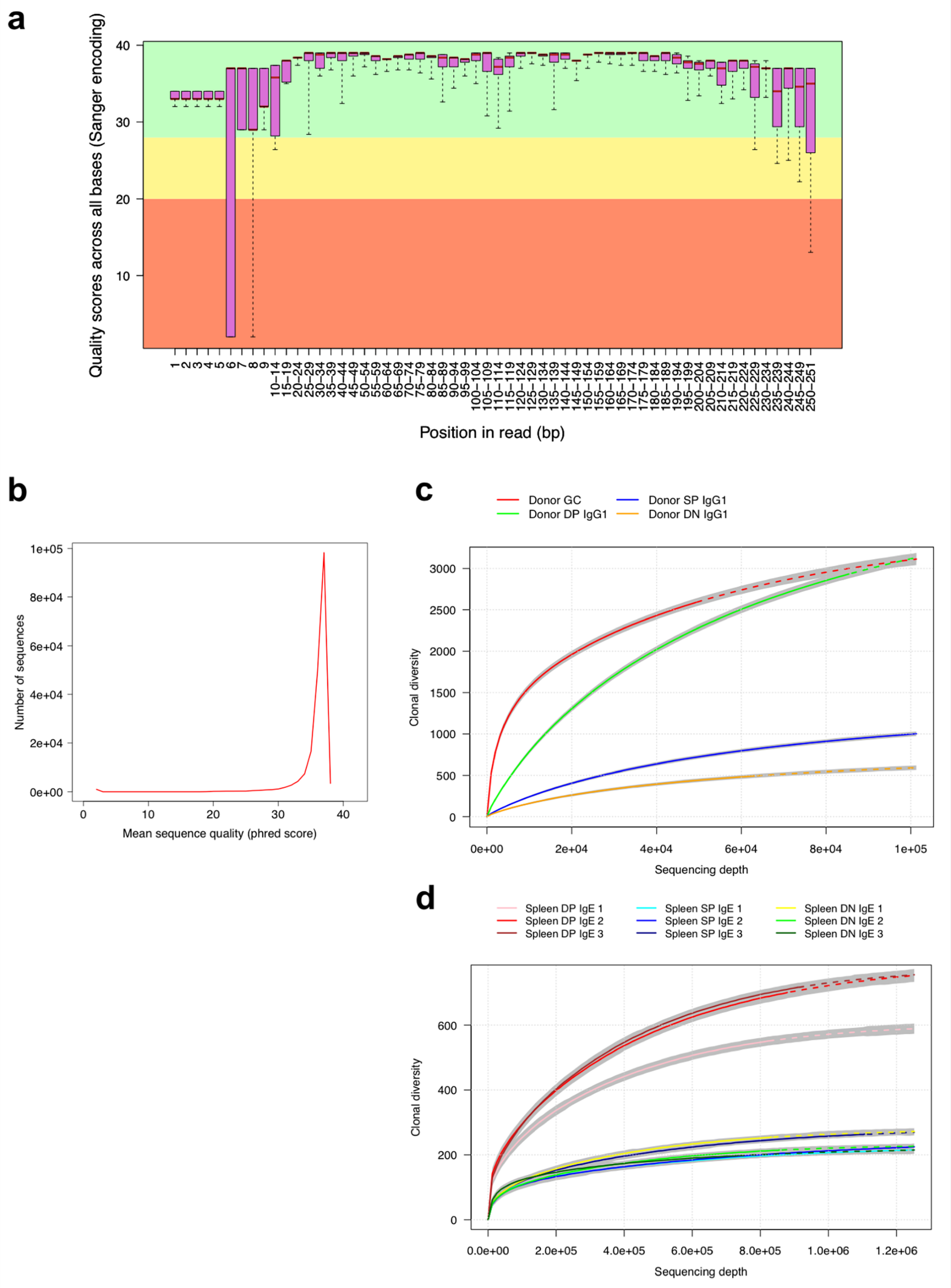

Supplementary Figure 4. VDJ H repertoire quality controls. (**a-b**) Sequence data quality. (**a**) Statistics of base quality scores on phred scale at various base positions in the sequencing reads; bp: base pairs. (**b**) Average base quality of entire reads. (**c-d**) Rarefaction curves of donor IgG1 MBC and IgG1 GC (**c**), and IgE sequences in recipients of DP, SP or DN IgG1 MBC (**d**). The solid portion of a curve is the observed data while the dotted portion is extrapolated by fitting a multinomial model.

Supplementary Figure 5

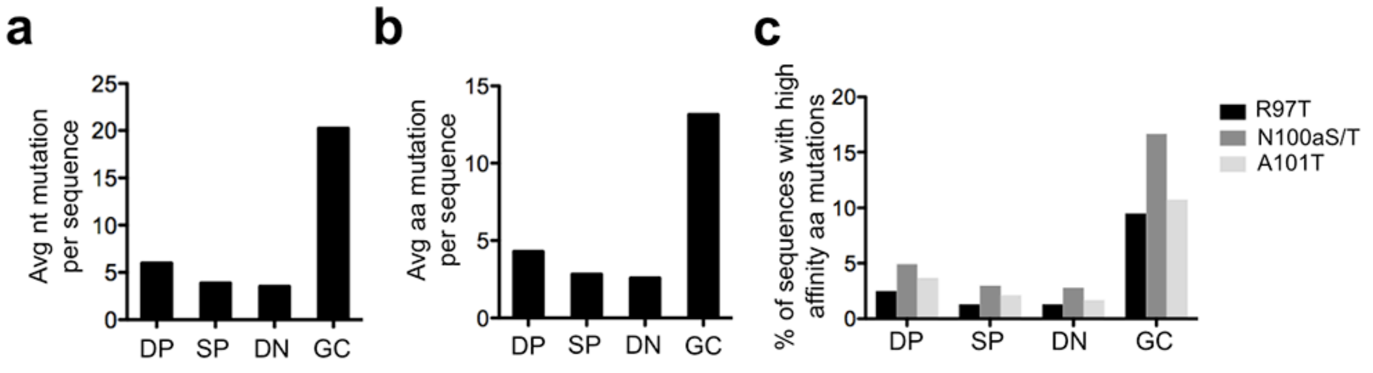

Supplementary Figure 5. VDJ H mutations in IgG1 MBC. Nucleotide (nt) and amino acid (aa) mutations were determined in the VDJ H genes of DP, SP and DN IgG1 MBC subsets, and in GC IgG1 cells isolated from TBmc mice 10 weeks after immunisation with OVA-PEP1. Average (Avg) number of nt (**a**) and aa (**b**) mutations per sequence. (**c**) Percentage of sequences containing R97T, N100aS/T or A101T CDR3 high affinity mutations. VDJ H repertoire sequencing was performed using RNAseq as described in the Methods and in Fig. 3. The data is representative of two independent experiments.

Supplementary Figure 6

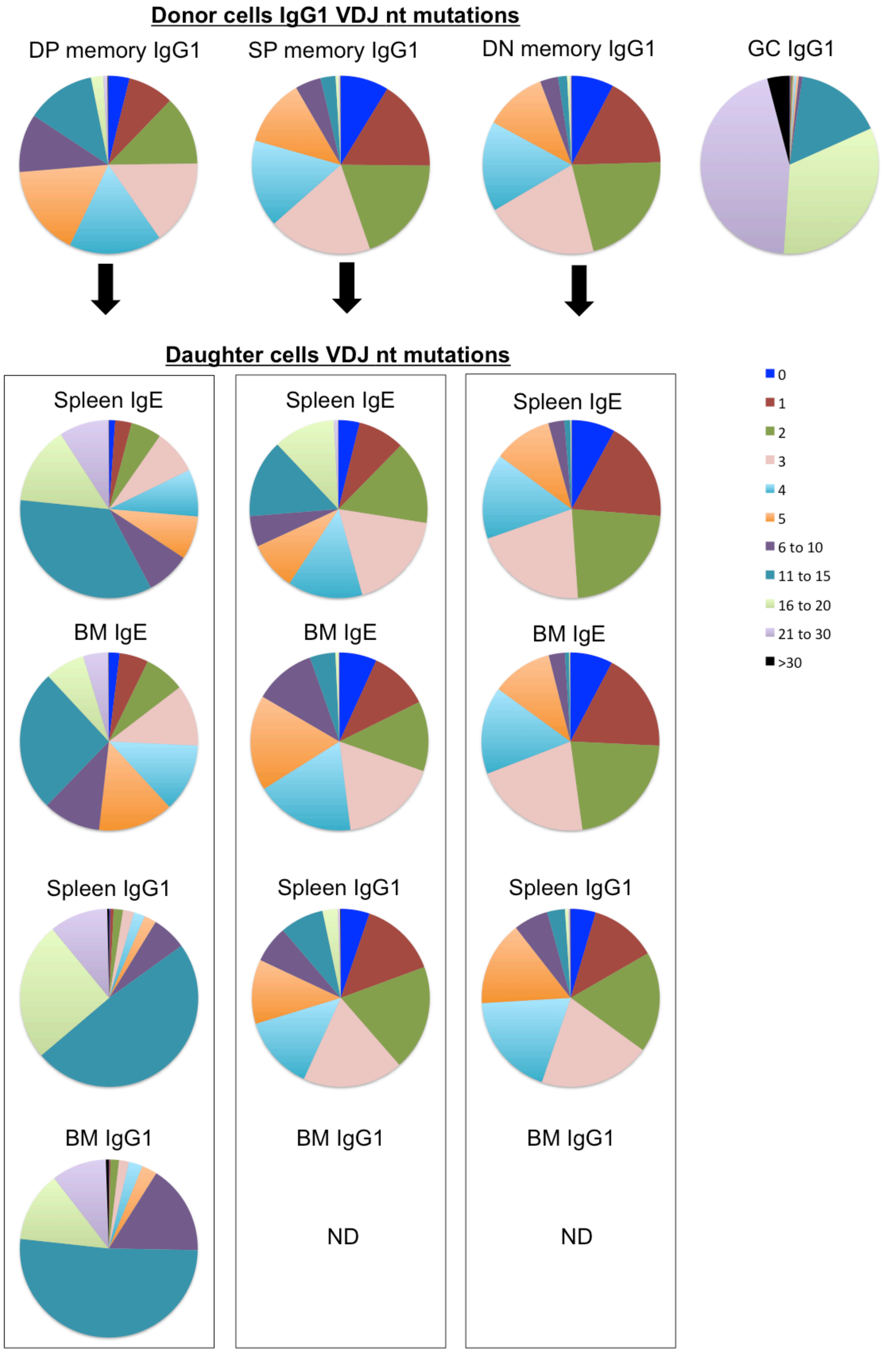

Supplementary Figure 6. Enrichment in VDJ H nucleotide mutations in IgE and IgG1 derived from DP IgG1 MBC. The pie charts show the frequency of VDJ H sequences with the indicated number of nucleotide (nt) mutations in parental DP, SP and DN IgG1 MBC, and their IgE and IgG1 progenies in spleen and BM of recipient mice 2 weeks after transfer/immunisation. Parental IgG1 MBC and GC sequences were obtained from single samples sorted from a pool of immunised mice. IgE and IgG1 daughter sequences were obtained and analysed for each individual recipient mouse. Data in the progeny (daughter) cells pie graphics are average of 3 mice and are representative of two independent experiments. ND: not detected.

Supplementary Figure 7

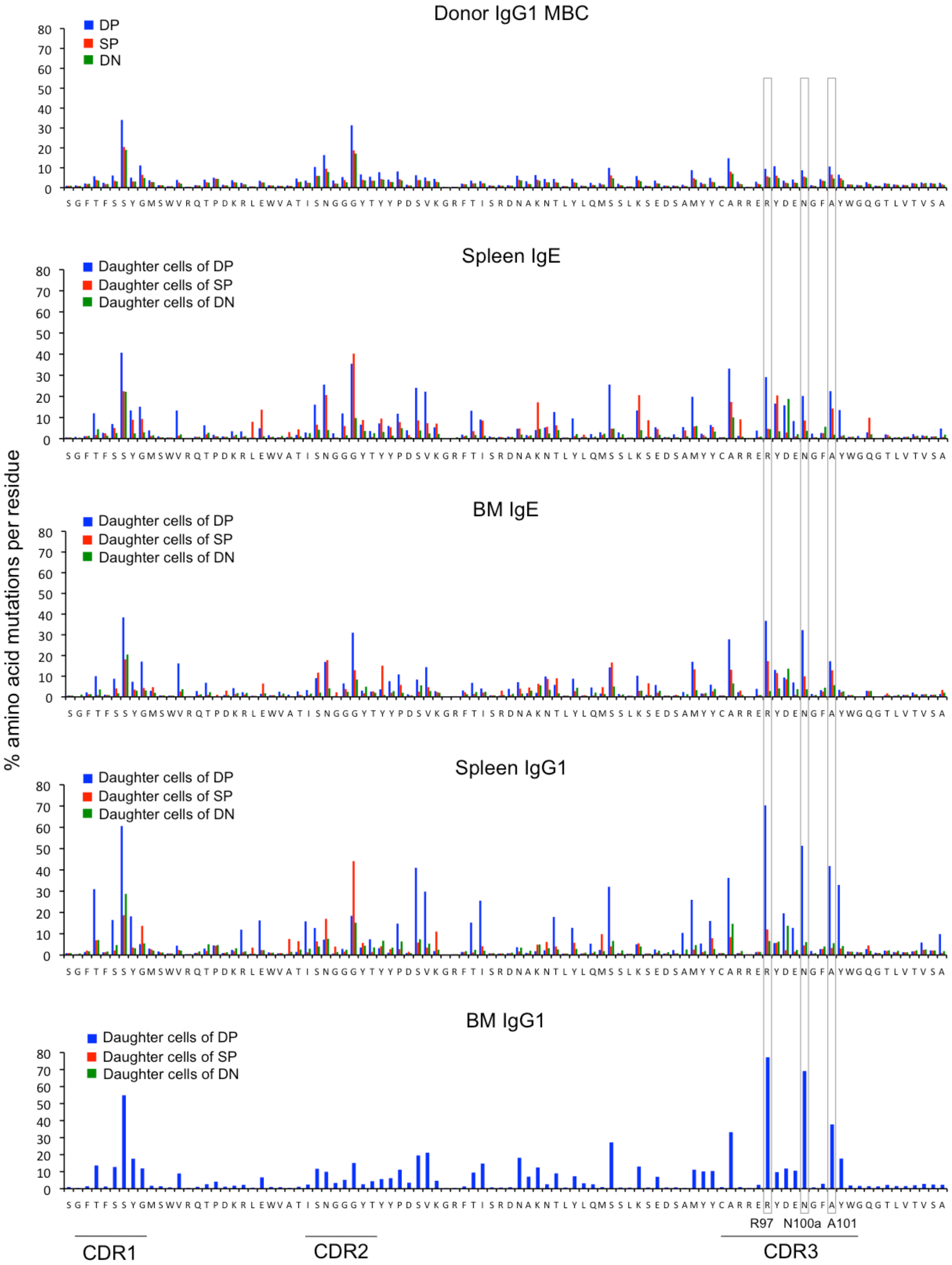

Supplementary Figure 7. CDR3 high affinity mutations are enriched in VDJ H IgE and IgG1 derived from DP IgG1 MBC. The bar graphs show the percentage of amino acid mutations per residue along the sequence of IgE and IgG1 VDJ H genes in donor DP, SP and DN IgG1 MBC and their IgE and IgG1 progenies in the spleen and BM of recipient mice. CDR1, CDR2 and CDR3 domains are indicated. The location of the three amino acid mutations in CDR3 that account for high affinity binding to PEP1 is shown. Data of daughter sequences are the average of 3 mice per group and are representative of two independent experiments.

Supplementary Figure 8

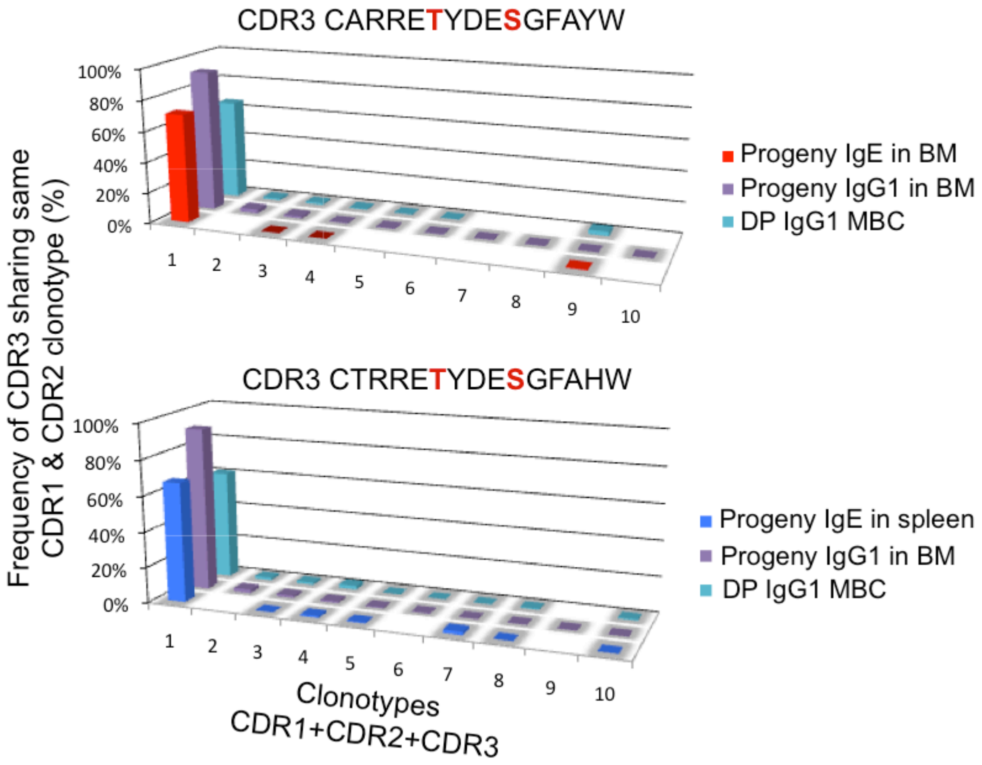

Supplementary Figure 8. High affinity PC are formed by clonal selection of existing memory precursors. Frequency of the top 10 CDR1, CDR2 and CDR3 sequence combinations for VDJ H genes containing either of the two indicated high affinity CDR3 sequences. Data for DP IgG1 MBC, and IgE BM and IgG1 BM progenies are shown for CDR3 sequence CARRE**T**YDE**S**GFAYW, and data for donor DP IgG1 MBC and IgE spleen and IgG1 BM progenies are shown for CDR3 CTRRE**T**YDE**S**GFAHW. The results were obtained from 1 recipient mouse and are representative of the analysis.

Supplementary Figure 9

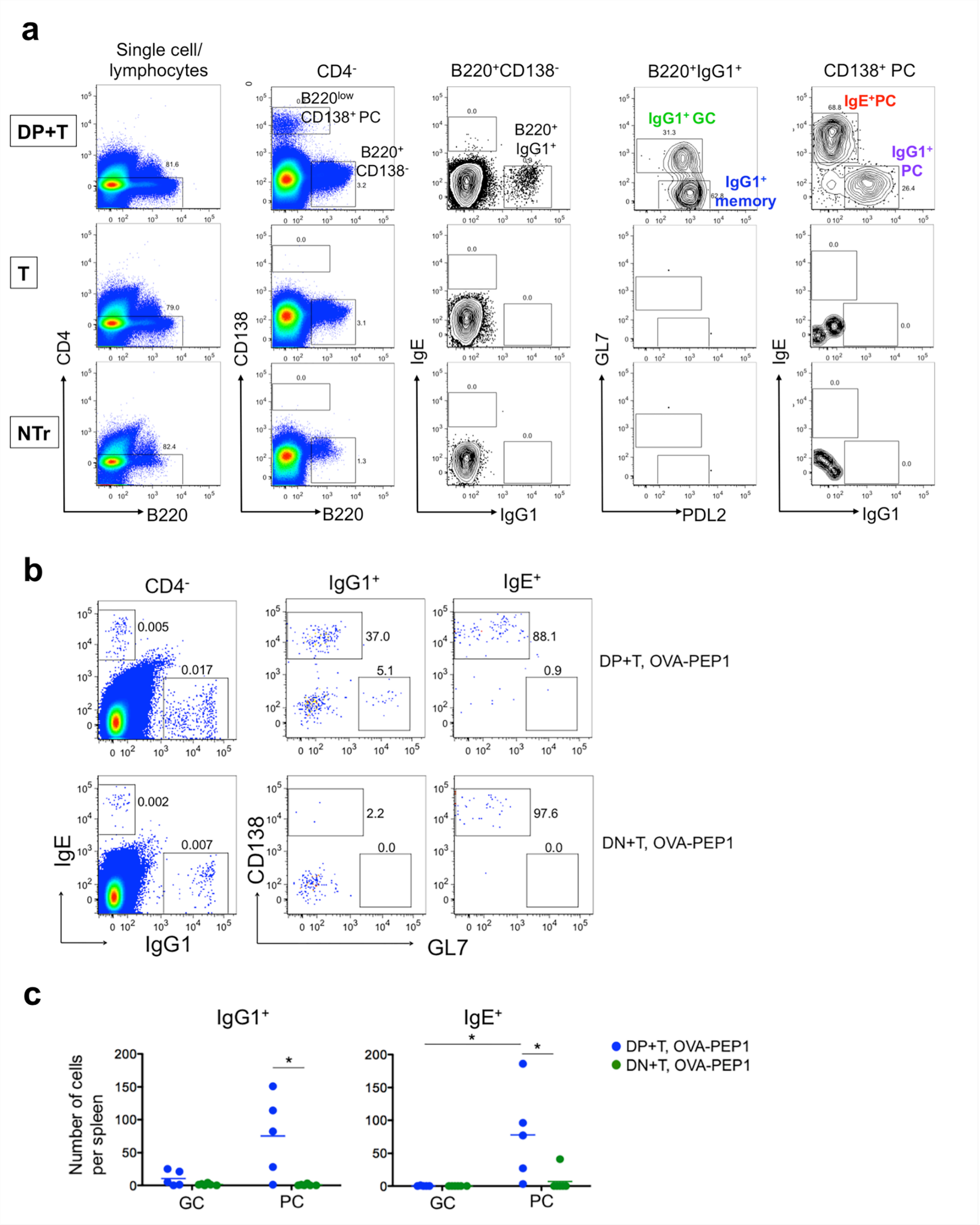

Supplementary Figure 9. CD73$^+$CD80$^+$ IgG1 MBC are poised to respond fast to activation. DP and DN IgG1 MBC were isolated from TBmc mice 10 weeks after immunisation with OVA-PEP1, and transferred together with CD4 memory T cells into *Rag1* KO recipients. Recipient mice were immediately immunised with OVA-PEP1. Donor-derived cells in the spleen of recipient mice were analysed after 1 and 6 weeks by flow cytometry (Fig. 5). (**a**) Gating strategy for flow cytometry analysis. Spleen cells were stained with antibodies to B220, CD4, CD138, GL7, PDL2, IgG1, IgE and the proliferation antigen Ki67. Dead cells were identified by DAPI staining. After single cell/lymphocyte gating using FSC/SSC parameters as described in **Supplementary Fig. 1a**, DAPI$^-$CD4$^-$ cells were identified (left row plots). B220^{low}CD138$^+$ cells and B220$^+$CD138$^-$ B cells were then gated among DAPI$^-$CD4$^-$ cells (second row from left). Among B220$^+$IgG1$^+$ cells (third and fourth rows from left), GC cells were distinguished by GL7 expression, and memory B cells by PDL2 expression. The plots on the right show IgE$^+$ and IgG1$^+$ cells within the CD138$^+$ PC gate. Histograms of Ki67 staining of gated cells (colour-labelled gates) are shown in **Fig. 5d**. The analysis shows one representative samples from the DP+T recipient group, a sample from a *Rag1* KO mouse transferred with only memory T cells (T), and a sample from a non-transferred *Rag1* KO mouse (NTr). (**b-c**) To compare early activation and differentiation of the DP and DN IgG1 MBC subsets, recipient mice were analysed 6 days after transfer/immunisation, the earliest time in which we consistently observed donor cell differentiation. Flow cytometry staining was performed using antibodies to B220, CD4, CD138, GL7, PDL2, IgG1, IgE. (**b**) Representative plots show the percentage of IgE$^+$ and IgG1$^+$ cells among CD4$^-$ cells (left plots), and the percentage of GC (GL7$^+$) cells and PC (CD138$^+$) among IgG1$^+$ (middle plots) and IgE$^+$ cells (right plots). (**c**) Number of IgG1$^+$ GC, IgG1$^+$ PC, IgE$^+$ GC and IgE$^+$ PC per spleen in recipient mice 6 days after transfer/immunisation. Each dot represents 1 recipient mouse. The averages of 5 samples (DP+T) and 6 samples (DN+T) are shown. Statistical analysis was performed using Mann-Whitney-Wilcoxon *U*-test. * $P<0.05$. The data is representative of three independent experiments.

Supplementary Figure 10

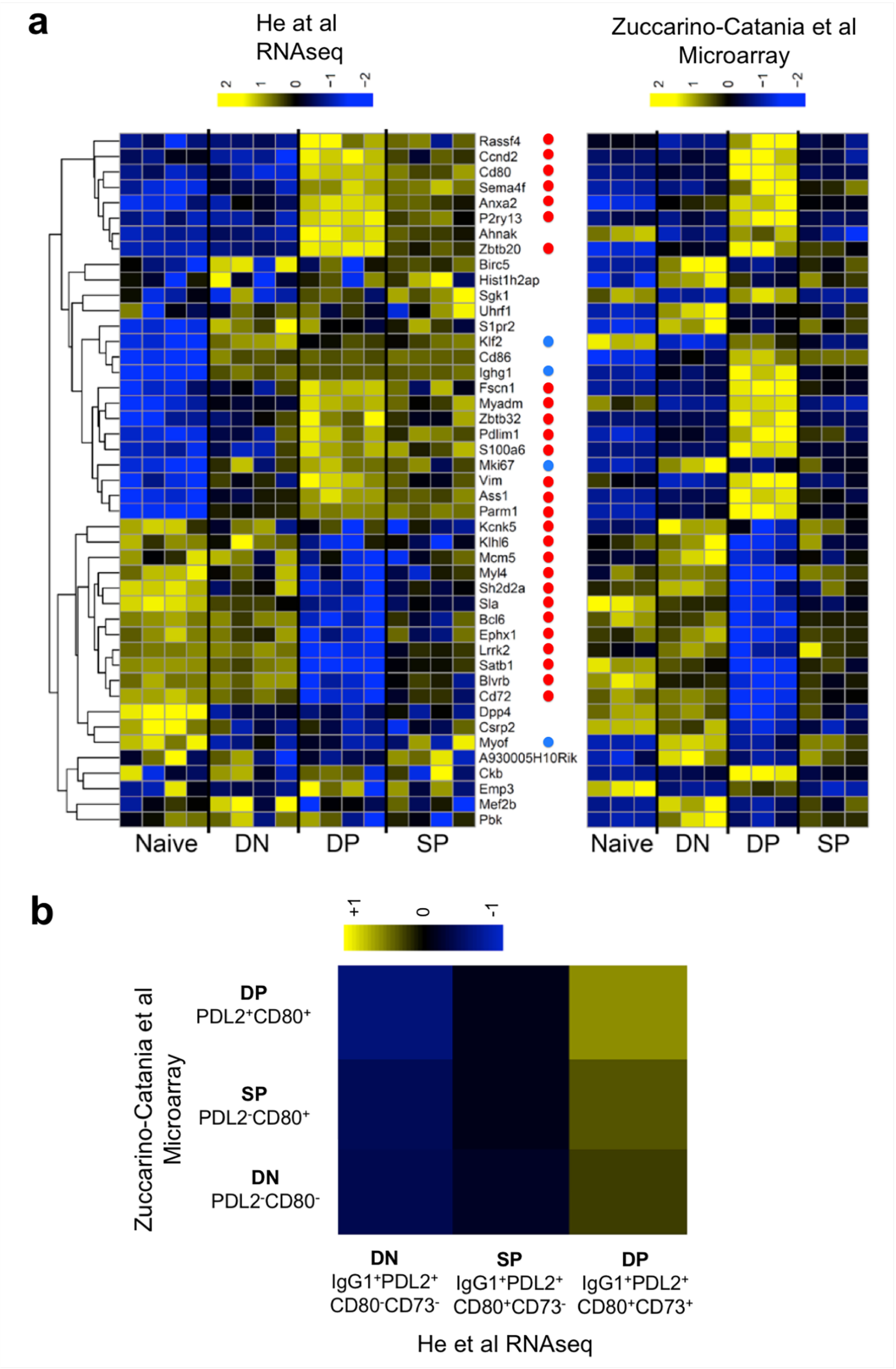

Supplementary Figure 10. Comparison of the RNAseq profile of IgG1 MBC subsets with a published microarray dataset[1]. (**a**) Heat map of the expression of the differentially expressed gene set identified by Zuccarino-Catania et al[1] (right) in our RNAseq dataset (left, He et al). Expression levels were normalised to Z-score on each row of the heat map. The memory populations analysed by us were sorted as follows: IgG1$^+$PDL2$^+$CD73$^+$CD80$^+$ (DP); IgG1$^+$PDL2$^+$CD73$^-$CD80$^+$ (SP); IgG1$^+$PDL2$^+$CD73$^-$CD80$^-$ (DN). The populations analysed by Zuccarino-Catania et al were sorted as follows: PDL2$^+$CD80$^+$ (DP); PDL2$^-$CD80$^+$ (SP); PDL2$^-$CD80$^-$ (DN). The difference in isotype composition between the two analysis is evident in the expression of *Ighg1* (encoding IgG1 constant region): *Ighg1* is uniform among our memory DP, SP and DN samples (He et al, this manuscript), while it is differentially expressed in the DP population in Zuccarino-Catania et al. Red dots indicate genes with similar regulation in the two studies. Blue dots mark genes with dissimilar regulation in the two studies. (**b**) CMap comparison of IgG1 MBC subset expression datasets from our RNAseq analysis with the MBC subset data from the Zuccarino-Catania et al microarray analysis.

Supplementary Figure 11

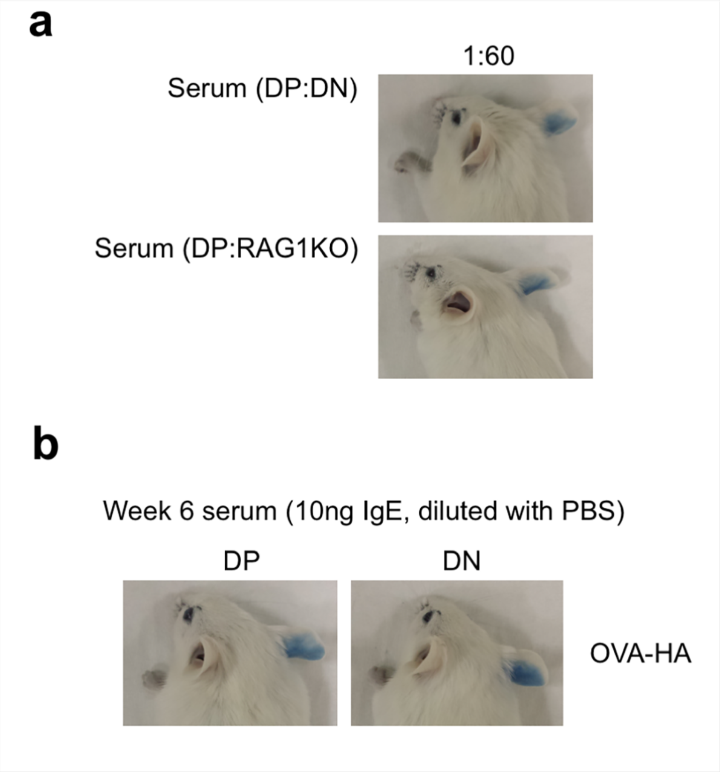

Supplementary Figure 11. Anaphylactic potential of IgE antibodies derived from DP and DN IgG1 MBC. DP and DN IgG1 MBC, and CD4 memory T cells, were obtained from PEP1-immunised TBmc mice and transferred into *Rag1* KO mice as described in **Fig. 2a**,**c-d**. The recipient mice were immunised with OVA-PEP1 and serum was collected 6 weeks after transfer/immunisation. Serum from each transferred group was depleted of IgG and tested in a passive cutaneous anaphylaxis (PCA) assay. (**a**) Serum from mice transfused with DP IgG1 MBC (DP serum) was diluted with serum from mice transfused with DN IgG1 MBC (DN serum) at 1:60 ratios. As control, DP serum was diluted with sera from untreated *Rag1* KO mice at 1:60 ratios. The results show that at a 60-fold excess, the DN serum did not inhibit anaphylaxis to PEP1 mediated by the DP serum. (**b**) To determine if IgE antibodies in the DN serum were functional, a PCA assay was carried out using OVA-HA as an antigen, since HA is recognised with high affinity by unmutated antibodies from TBmc mice[2]. IgG depleted sera from recipient mice diluted with PBS to contain 0.5ng/ml of IgE were injected in the ears of mice. The positive PCA mediated by DN serum demonstrates that IgE antibodies in the DN serum bind to mast cells and can mediate degranulation if crosslinked. For the PCA assays in, 20 µl of diluted serum were injected intradermal into the ears of BALB/c mice. 24 hours later, 50 µg of OVA-PEP1 (**a**) or OVA-HA (**b**) in PBS were injected intravenously together with 1% Evans blue. 30 minutes later the mice were sacrificed, and the extent of Evans blue extravasation was determined visually.

Supplementary Table 1

Most CDR3 sequences containing high affinity mutations are found in the parental DP IgG1 MBC and their IgE and IgG1 progenies.

		PROGENY				DONOR
hi aff CDR3 ID	high affinity CDR3 sequencies#	IgE spleen	IgE BM	IgG1 Spleen	IgG1 BM	DP IgG1 MBC
s1	CARRETYDESGFTYW	P	P	P	P	P
s2	CSRRETYDESGFTYW	P	P	P	P	P
s3	CTRRETYDESGFTFW	P	P	P	P	P
s4	CARRETYDESGFAYW	P	P	P	P	P
s5	CTRRETYDESGFAYW	P	P	P	P	P
s6*	CTRRETIDESGFAYW	P	P	A	A	A
s7	CARRETYDESGFAHW	P	P	P	P	P
s8	CSRRETYDESGFAYW	P	P	P	P	P
s9	CTRRETYDESGFAHW	P	P	P	P	P
s10	CTRRETFDESGFAYW	P	P	P	P	P
s11	CTRRETSDESGFAHW	P	P	P	P	P
s12*	CSRRETYDESGFVYW	P	P	A	A	A
s13	CARRETYDENGFTYW	P	P	P	P	P
s14	CARRETYGENGFTHW	P	P	P	P	P
s15	CTRRETYDERGFTYW	P	P	P	P	P
s16	CARRETYDENGFAYW	P	P	P	P	P
s17	CARRETFDENGFAYW	P	P	P	P	P
s18	CARRETYDEHGFAYW	P	P	P	P	P
s19	CARRETNDENGFAYW	P	P	P	P	P
s20	CTRRETYDEHGFAYW	P	P	P	P	P
s21	CARRETFDEHGFAYW	P	P	P	P	P
s22	CARRERYDESGFAYW	P	P	P	P	P
s23	CTRRERYDESGFAYW	P	P	P	P	P
s24	CAGRERYDESGFAYW	P	P	P	P	P
s25	CARRRRYDESGFAYW	P	P	A	A	P
s26	CARRERYDVSGFAYW	P	P	P	P	P
s27	CARRENYDGTGFAYW	P	P	P	P	P
s28	CARRERFDETGFASW	P	P	P	A	P
s29	CTRRGRYDETGFVYW	P	P	P	P	P
s30	CSRRGKYDENGFTSW	P	P	P	P	P
s31	CARRERYDENGFTYW	P	P	P	P	P
s32	CTRRERYDENGFTYW	P	P	P	P	P
s33	CARRERYDENRFTYW	P	P	P	P	P
s34	CARRESYDENGFTYW	P	P	P	P	P
s35	CARRESYDERGFTYW	P	P	P	P	P

Letters in red indicate high affinity amino acid mutations.
P: present, for progeny it indicates that the sequence was found in at least one of the 3 samples.
A: absent, for progeny samples it indicates that the sequence was absent from all 3 samples.
* indicate sequences not found in the donor populations.

Supplementary Table 2

Inheritance of silent mutations supports selection of high affinity memory clones

	CDR3 Sequence 2#	DONOR	PROGENY	
aa	C A R R E T Y D E S G F A Y W	DP IgG1 MBC (cc)	BM IgG1 M3 (cc)	BM IgE M3 (cc)
nt	TGTGCAAGACGGGAGACGTATGACGAGAGCGGGTTTGCTTACTGG	357	163419	42675
nt	TGTGCAAGACGGGAAACATACGACGAGAGCGGGTTTGCTTACTGG	239	98219	771
nt	TGTGCAAGACGGGAGACGTACGACGAGAGCGGGTTTGCTTACTGG	1681	6178	26446
nt	TGTGCAAGACGGGAGACATACGACGAGAGCGGGTTTGCTTACTGG	239	2748	131
nt	TGTGCAAGACGGGAGACTTACGACGAGAGCGGGTTTGCTTACTGG	1109	1271	0
nt	TGCGCAAGACGGGAGACGTATGACGAGAGCGGGTTTGCTTACTGG	4	1202	215
nt	TGTGCAAGACGGGAAACGTACGACGAGAGCGGGTTTGCTTACTGG	87	1163	0
nt	TGTGCGAGACGGGAGACGTATGACGAGAGCGGGTTTGCTTACTGG	4	938	194
nt	TGTGCAAGGCGGGAGACGTATGACGAGAGCGGGTTTGCTTACTGG	2	936	258
nt	TGTGCAAGACGGGAGACGTATGACGAGAGCGGGTTTGCCTACTGG	4	774	0
nt	TGCGCAAGACGGGAAACATACGACGAGAGCGGGTTTGCTTACTGG	3	633	0
nt	TGTGCAAGGCGGGAAACATACGACGAGAGCGGGTTTGCTTACTGG	3	584	5
nt	TGTGCGAGACGGGAAACATACGACGAGAGCGGGTTTGCTTACTGG	2	482	0
nt	TGTGCAAGACGGGAGACGTACGACGAGAGCGGATTTGCTTACTGG	165	100	0
nt	TGTGCAAGGCGGGAGACGTACGACGAGAGCGGGTTTGCTTACTGG	17	53	143
nt	TGTGCGAGACGGGAGACGTACGACGAGAGCGGGTTTGCTTACTGG	12	29	156
nt	TGCGCAAGACGGGAGACGTACGACGAGAGCGGGTTTGCTTACTGG	0	29	180
WT nt	TGTGCAAGACGGGAGA**GG**TACGACGAGA**A**CGGGTTTGCTTACTGG			
WT aa	C A R R E **R** Y D E **N** G F A Y W			

Distribution of encoding nucleotide sequences for one CDR3 amino acid sequence containing high affinity mutations (CARRETYDESGFAYW, sequence 2 in Figure 4A). High affinity amino acid mutations and encoding nucleotide mutations are in red. Silent mutations are in blue.
aa: amino acid; nt: nucleotide; cc: number of reads.
M3: One representative mouse of 3 recipient mice.

Supplementary Table 3

Antibodies used in flow cytometry and ELISA

Antibody target	Clone/origin	Conjugation	Source	Working dilution
CD16/CD32	93	Unconjugated	eBioscience	10 µg/ml
Ki67	16A8	PE	BioLegend	1:600
IgE	R1E4	Alexa Fluor 647	In house	1:800
CD3e	145-2C11	Biotin	eBioscience	1:200
IgD	11-26c	Biotin	eBioscience	1:200
CD138	281-2	Biotin	BD Bioscience	1:200
CD138	281-2	Brilliant Violet 510	BD Bioscience	1:200
TER119	TER-119	Biotin	eBioscience	1:200
IgM	II/41	eFluor450	eBioscience	1:100
B220	RA3-6B2	APC-eFluor780	eBioscience	1:200
IgG1	M1-14D12	PE-Cy7	eBioscience	1:400
CD80	16-10A1	PE	eBioscience	1:200
CD80	16-10A1	PE-CF594	BD Bioscience	1:400
CD73	TY/11.8	PerCP-eFluor710	eBioscience	1:200
PDL2	TY25	BUV395 or PE	BD Bioscience	1:200
GL7	GL7	APC or FITC	BD Bioscience	1:200
GL7	GL7	eFluor 450	eBioscience	1:200
CD4	RM4-5	eVolve605	eBioscience	1:50
CD4	GK1.5	Alexa Fluor 700	eBioscience	1:200
CD45RB	C363.16A	APC	eBioscience	1:200
CD25	PC61.5	PerCP	eBioscience	1:200
IgE	LO-ME-3	Unconjugated	Invitrogen	2 µg/ml
IgE	Goat-anti-mouse IgE	HRP	Southern Biotech	1:2000
IgEa	UH297	biotin	BioLegend	1:2000
IgG1	Goat $F(ab')_2$-anti-mouse IgG1	Unconjugated	Southern Biotech	2 µg/ml
IgG1	Goat-anti-mouse IgG1	biotin	Southern Biotech	1:2000
IgG1a	10.9	biotin	BD Bioscience	1:2000

Supplementary References

1. Zuccarino-Catania GV, Sadanand S, Weisel FJ, Tomayko MM, Meng H, Kleinstein SH*, et al.* CD80 and PD-L2 define functionally distinct memory B cell subsets that are independent of antibody isotype. *Nat Immunol* 2014, **15**(7)**:** 631-637.

2. Erazo A, Kutchukhidze N, Leung M, Christ AP, Urban JF, Jr., Curotto de Lafaille MA*, et al.* Unique maturation program of the IgE response in vivo. *Immunity* 2007, **26**(2)**:** 191-203.
