# Supplementary material for: IgG1 memory B cells keep the memory of IgE responses
Source: Nat Commun. 2017 Sep 21;8:641. doi: 10.1038/s41467-017-00723-0 (PMC5608722; doi:10.1038/s41467-017-00723-0)
Supplement: Supplementary file 2 — Description of Additional Supplementary Files [file 41467_2017_723_MOESM2_ESM.pdf]

Description of Additional Supplementary Files

File name: Supplementary Data 1
Description: The clonal relationships between high affinity CDR3-VDJ H genes in parental DP IgG1 MBP and their IgE and IgG1 progenies were analyzed. For this, the presence and frequency of each nucleotide sequence encoding a CDR3 domain containing high affinity amino acid residues was determined. The 35 high affinity-CDR3 amino acid sequences are also described in **Supplementary Table 1**. The Supplementary data 1a sheet shows all high affinity CDR3 amino acid sequences and all encoding nucleotide sequences found in the dataset, and their frequency in IgE and IgG1 progenies and in the parental donor DP IgG1 MBC. **Supplementary data sheets 1b** and **1c** display the distribution of high affinity IgE (**1b**) and IgG1 (**1c**) CDR3 nucleotide encoding sequences in each recipient mouse and the percentage of those sequence that were present in the donor DP IgG1 MBC. These data is summarized in **Fig. 4c**.

File name: Supplementary Data 2
Description: Transcriptional gene expression differences between DP, SP and DN IgG1 MBC subsets were determined by RNA sequencing. Differentially expressed genes (DEG) across cell types were defined in pairwise comparisons and are shown in **Supplementary data sheets 2a** (DP vs DN), **2b** (DP vs SP) and **2c** (SP vs DN). Data sheet **2d** displays the combined DEG list.

File name: Supplementary Data 3
Description: Differentially expressed genes (DEG) from the comparison of the RNAseq datasets of DP, SP and DN IgG1 MBC subsets were analyzed using gene ontology (GO) enrichment and the results are shown in **Supplementary data sheet 3a**. Heatmaps for GO-identified DEG enriched for transcriptional regulation, cellular receptors and kinases are shown in **Fig. 6c-e**. Differentially modulated molecular pathways and biological processes identified using Metacore are shown in **data sheets 3b** and **3c** respectively.
